# Soluble Receptor for Advanced Glycation End-products regulates age-associated Cardiac Fibrosis

**DOI:** 10.7150/ijbs.56379

**Published:** 2021-06-11

**Authors:** Francesco Scavello, Filippo Zeni, Giuseppina Milano, Federica Macrì, Stefania Castiglione, Estella Zuccolo, Alessandro Scopece, Giovanni Pezone, Calogero C. Tedesco, Patrizia Nigro, Genny Degani, Elisa Gambini, Fabrizio Veglia, Laura Popolo, Giulio Pompilio, Gualtiero I. Colombo, Marco E. Bianchi, Angela Raucci

**Affiliations:** 1 Unit of Experimental Cardio-Oncology and Cardiovascular Aging, Centro Cardiologico Monzino-IRCCS, Milan, Italy.; 2 Vascular Biology and Regenerative Medicine Unit, Centro Cardiologico Monzino-IRCCS, Milan, Italy.; 3 Unit of Biostatistics, Centro Cardiologico Monzino-IRCCS, Milan, Italy.; 4 Department of Biosciences, University of Milan, Milan, Italy.; 5 Unit of Immunology and Functional Genomics, Centro Cardiologico Monzino-IRCCS, Milan, Italy.; 6 Chromatin Dynamics Unit, San Raffaele University and IRCCS San Raffaele Hospital, Milan, Italy.

**Keywords:** sRAGE, aging, fibrosis, heart failure, cardiac remodeling

## Abstract

Myocardial aging increases the cardiovascular risk in the elderly. The Receptor for Advanced Glycation End-products (RAGE) is involved in age-related disorders. The soluble isoform (sRAGE) acts as a scavenger blocking the membrane-bound receptor activation. This study aims at investigating RAGE contribution to age-related cardiac remodeling.

We analyzed the cardiac function of three different age groups of female *Rage-/-* and C57BL/6N (WT) mice: 2.5- (Young), 12- (Middle-age, MA) and 21-months (Old) old. While aging,* Rage-/-* mice displayed an increase in left ventricle (LV) dimensions compared to age-matched WT animals, with the main differences observed in the MA groups. *Rage-/-* mice showed higher fibrosis and a larger number of α-Smooth Muscle Actin (SMA)+ cells with age, along with increased expression of pro-fibrotic Transforming Growth Factor (TGF)-β1 pathway components. RAGE isoforms were undetectable in LV of WT mice, nevertheless, circulating sRAGE declined with aging and inversely associated with LV diastolic dimensions. Human cardiac fibroblasts stimulated with sRAGE exhibited a reduction in proliferation, pro-fibrotic proteins and TGF-beta Receptor 1 (TGFbR1) expression and Smad2-3 activation. Finally, sRAGE administration to MA WT animals reduced cardiac fibrosis.

Hence, our work shows that RAGE associates with age-dependent myocardial changes and indicates sRAGE as an inhibitor of cardiac fibroblasts differentiation and age-dependent cardiac fibrosis.

## Introduction

The exponential increase of cardiovascular (CV) diseases (CVD) in the elderly population, including coronary artery disease (CAD), left ventricular hypertrophy and heart failure (HF), is largely ascribable to the long-term exposure to CV risk factors present in later life, like hypertension and diabetes. However, intrinsic cardiac aging is *per se* a risk factor and, by making the heart more vulnerable to stress, contributes to increased CV mortality and morbidity. During aging, the heart undergoes anatomical changes characterized by alterations in volume and stiffness, attributable to cardiomyocytes (CM) hypertrophy and interstitial fibrosis, with consequent decline in the diastolic function 1-3. The activation and proliferation of cardiac fibroblasts (CFs), the main regulators of extracellular matrix (ECM) turnover, are the prominent cause of the progressive myocardial fibrosis occurring during aging 4. The identification of protective molecules that allow the heart to “age slowly” could lead to new therapeutic approaches reducing the risk of CVD onset.

The Receptor for Advanced Glycation End-products (RAGE) is a pattern recognition receptor (PRR) present at very low levels in most tissues in homeostasis, with the exception of the lung, which exhibits high basal expression of RAGE 5, 6. The membrane-bound full-length RAGE (FL-RAGE) is composed of three extracellular Ig-like domains, a single transmembrane helix and a short cytosolic tail, and can bind several ligands, such as Advanced Glycation End-products (AGEs), S100 proteins, High Mobility Group Box-1 (HMGB1) and ECM proteins 7-9. Most RAGE ligands bind other receptors as well. Binding of FL-RAGE to its ligands activates signaling pathways, including MAPKs and NF-κB, and promotes inflammation, cell migration and adhesion in the midst of pro-inflammatory diseases 9-13.

FL-RAGE undergoes proteolytic shedding by ADAM10 or matrix metalloproteinases (MMPs) to produce the cleaved soluble RAGE (cRAGE) form 14. Furthermore, the RAGE primary transcript is subject to alternative splicing that generates species- and tissue-specific isoforms 15-17. hRAGE and mRAGE are the most abundant variants encoding for FL-RAGE in human (h) and mouse (m), respectively; hRAGE_v1 and mRAGE_v1/v3 encode the secreted soluble esRAGE protein 15-17. The rodent-specific mRAGE_v4 variant produces the short membrane-bound protein M-RAGE that is resistant to ADAM10 proteolysis 15. Collectively, cRAGE and esRAGE constitute the soluble RAGE (sRAGE) fraction found in human circulation 18; they appear functionally equivalent, acting as scavengers that neutralize RAGE ligands 14. Indeed, in several experimental animal models, administration of sRAGE exerts protective action by limiting inflammation associated to lung diseases, atherosclerosis, diabetes and tumor progression 19-23.

Human studies report decreased levels of circulating sRAGE or esRAGE in patients with hypertension 24, in the early stage of type 2 diabetes and obesity 25, 26. In CVD-free subjects, sRAGE negatively correlates with carotid artery intima-media thickness progression 27. On the other hand, higher levels of sRAGE are often associated with established CVD and end-stage diseases 28. In a healthy population cRAGE decreases with age whereas esRAGE correlates with obesity and insulin resistance markers 18 and centenarians have a more elevated sRAGE amount than young subjects 29. Therefore, high levels of sRAGE could be a marker of successful aging and the two soluble isoforms could function as early predictors of different CV risk factors.

RAGE contribution to aging-associated decline of organ function has been scarcely investigated. Old *Rage-/-* mice spontaneously develop pulmonary fibrosis with senescent lesions and show higher frequency of lung carcinoma 30, 31. Hence, RAGE could be a regulator of organ aging; however, its role in intrinsic myocardial aging has never been explored. Here, we investigated for the first time RAGE's contribution to aging-dependent cardiac remodeling. RAGE deficiency in mice accelerated age-induced left ventricle (LV) dimension changes and fibrosis and induced TGF-β1 signaling without affecting LV systolic function and inflammation. Interestingly, circulating sRAGE decreased with age and inversely correlated with LV diastolic dimensions. Recombinant sRAGE reduced the proliferation of human cardiac fibroblasts (hcFbs) and their trans-differentiation into myofibroblast (MyoFbs) *in vitro* and, consistently, its administration in adult WT mice reduced age-dependent fibrosis.

Our findings demonstrate that RAGE is associated with age-dependent cardiac remodeling and the progression of cardiac fibrosis. More specifically, our work indicates circulating sRAGE as an inhibitor of cFbs transition into MyoFbs and suggests it as a feasible biomarker of cardiac aging. Thus, sRAGE may represent a promising molecule for future therapeutic strategies aimed at reducing cardiac fibrosis and promoting healthy aging.

## Material and Methods

### Animals

Female C57BL/6N WT (Charles River Laboratories, Calco, Italy) or *Rage-/-* mice 32 were housed in standard cages and fed a normal chow diet. Ten weeks-, twelve or twenty-one months-old (Young, Middle-age (MA) and Old, respectively) animals were generated, weighted and anesthetized with an intraperitoneal (i.p.) injection of ketamine-medetomidine cocktail (100 mg/Kg -10 mg/Kg) for blood collection and euthanized by exposure to carbon dioxide (>70%). Mice were perfused with phosphate-buffered saline from the apex of the heart that was dissected out along with other organs such as lung, brain, kidney, spleen and muscle and immediately frozen and processed randomly as described below. Length of tibia (TL) was also measured.

For the administration of recombinant murine sRAGE, 9 females MA C57BL/6N WT mice were randomly assigned to sRAGE (n=5) and control (CTRL; n=4) groups. Animals were daily i.p. injected with about 22 µg of sRAGE or equal volume of saline solution (CTRL) for eight consecutive days and, then, sacrificed as described above.

All procedures involving animals were performed in accordance with our Institutional Guidelines, which comply with national (D.L. n.116, G.U. suppl. 40, 18 February 1992) and international laws (EU Directive 2010/63/EU). The study was authorized by the National Ministry of Health-University of Milan Committee (Approval number 12/12-30012012).

The ELISA kit for mouse RAGE (#MRG00; R&D Systems Inc., Minneapolis, MN, USA) was used to test sRAGE in the serum of mice following manufacturer's instruction.

### Two-dimensional Echocardiography

Transthoracic echocardiography was performed using a Vevo 2100 high-resolution imaging system (Visual Sonics, Toronto, ON, Canada) and a 40-MHz linear transducer with simultaneous ECG recording. Analyses were performed on mice anesthetized with a mix of 1-1.5% isoflurane/70% N2O-30% O2 mix at 480-550 beats/min. Two-dimensional short-axis M-mode was recorded at the level of mid-papillary muscle to measure LV mass, LVESD, LVEDD, LVESV, LVEDV, LVEF LVFS, SV and LVPW, d. Data were analyzed with VisualSonics Cardiac Measurements Package.

### Immunohistochemistry

Isolated hearts were fixed in paraformaldehyde, embedded in paraffin and sectioned (6 μm). Heart sections were de-paraffinized, re-hydrated and boiled for 20 minutes in Dako Target Retrieval Solution Citrate pH 6 (#S2369, Glostrup, Denmark). After washing in PBS-0.1% Triton X-100 (PBS-T), slides were incubated in 3% H_2_O_2_ (Sigma-Aldrich) for 10 min and blocked in 5% goat serum in PBS-T for 1 hour (h) at room temperature (rt). Primary antibodies for Collagen I (5 µg/ml; #Ab292, Abcam, Cambridge, United Kingdom) CD45 (2 µg/ml; #Ab10558, Abcam), α-SMA (1 µg/ml; #ab5694, Abcam), Smad2-3 (1:100; #D7G7, Cell Signaling Technology, Leiden, The Netherlands) and TGFbetaR1 (2 µg/ml; #HPA056473, Sigma, St. Louis, MO, USA) were dissolved in antibody diluent (DAKO) or 1% goat serum in PBS-T and incubated overnight (o.n.) at 4 °C. A negative control in which the tissue was incubated with 1% goat serum PBS-T without the primary antibody was also included. Then, sections were incubated with biotin-conjugated goat anti-rabbit antibody (1:200; #BA-1000, Vector Laboratories, Burlingame, CA, USA) and, then with horseradish peroxidase (HRP)-conjugated streptavidin (ABC kit; PK-6100, Vector Laboratories) for 30 min at rt. Immunoreactions were revealed using 3.3'-Diaminobenzidine (ImmPACT DAB substrate, SK-4105, Vector Laboratories) as chromogen and slides were counterstained with hematoxylin. Images were taken by Axioskop II microscope (Zeiss, Oberkochen, Germany) using a digital camera (AxioCam Color-Zeiss) with the 20x objective. Quantification of Collagen I, α-SMA, nuclear Smad2-3 and TGFbetaR1 signal was determined on 5-10 different fields from each section using Axiovision SoftwareTM Rel 4.8 (Zeiss) considering the percentage of positive area defined as the ratio between the specific antigen positive area to the total area of the field. For CD45 staining, the number of positive cells/field (area) was indicated.

Fibrosis was assessed with a Masson's Thricrome staining kit according to the manufacture's instruction (# 04-010802, Bio-Optica, Milan, Italy). Images were taken with Axioskop II microscope (Zeiss) using a digital camera (AxioCam Color-Zeiss) with the 20x objective. Percentage of positive area was determined on 5-10 different fields from each section using Axiovision Software TM Rel 4.8 (Zeiss) considering the percentage of positive area defined as the ratio between the stained positive area to the total area of the field.

### Evaluation of cardiomyocytes hypertrophy

#### Wheat Germ Agglutinin (WGA) staining

Slides were incubated with orange-fluorescent tetramethylrhodamine conjugate WGA (2µg/ml; #W7024, Invitrogen, Carlsbad, CA, USA) for 10 min at rt and then with Hoechst 33342 (Life technologies, Carlsbad, CA, USA) for nuclei staining. Image acquisition was performed on optical fluorescent microscopy (APOTOME, Zeiss, Oberkochen, Germany) with the 40× objective. Cardiomyocytes (CM) cross sectional area (approximately 160 cells/group) was measured using AxioVision 4.8 software (Zeiss) considering those CM with the nucleus in the center of the cell surface 33.

#### Measure of isolated murine CM area

CMs were isolated from hearts of MA WT or *Rage-/-* mice with a Langendorff apparatus. All solutions were calibrated at 37 °C and saturated with 5% CO2/ 95% O2. Hearts were first perfused with the Perfusion Buffer (PB; 120 mM NaCl, 14.7 mM KCl, 0.6 mM KH_2_PO_4_, 0.6 mM Na_2_HPO_4_, 1.2 mM MgSO_4*_7H_2_O, 10 mM NaHEPES, 4.6 mM NaHCO_3_, 30 mM taurine, 10 mM 2,3-butanedione-monoxime, 5.5 mM Glucose; pH 7.4) for 4 minutes, and then with the Digestion Buffer [DB; PB containing 600 U/ml collagenase II (Worthington, Lakewood, NJ, USA)] for 3 minutes. The perfusion was continued with DB supplemented with 0.12 mM CaCl2 (DB2) for 8 minutes. Then, hearts were transferred to a 60-mm dish containing DB2 and cut into small pieces and further homogenized by gently pipetting after the addition of a Stopping Buffer (SB; PB with 10% FBS and 0.12 mM CaCl_2_). Cell suspension was passed through a 210 μm-nylon filter and let sediment by gravity for 10 minutes at 37 °C. CM pellet was resuspended in SB supplemented with 0.9 mM CaCl_2_ and let sediment for 14 minutes. Cells were resuspended in complete Minimun Essential Medium [MEM, with 10000 U/ml Penicillin, 10000 μg/ml Streptomycin (Sigma-Aldrich, St. Louis, MO, USA) and 20 mM L-Glutamine (Sigma-Aldrich)] containing 2.5% FBS and plated for 2 h. Then, medium was replaced with complete MEM containing 1% FBS and cultured up to 48 h. Images of CM were taken with optical microscopy (APOTOME, Zeiss) and area, major and minor diameters of 50-120 cells for each heart were measured using AxioVision 4.8 software (Zeiss).

### Production of recombinant sRAGE

Human VC1 domain of sRAGE was produced in *Pichia pastoris* as already described in ref 34. For the production of murine sRAGE, the recombinant pHIL-S1-mouse VC1-His plasmid directing the expression in *Pichia pastoris* of the VC1 domain spanning amino acid 23 to 231 of mouse RAGE, and harboring a C-terminal 6xHistidine tag, was obtained as previously described 34. The KM71 strain (his4, aox1::ARG4, arg4) of *Pichia pastoris* was transformed with the linearized pHIL-S1-mouseVC1-His expression cassette using the “EasyComp” chemical transformation method (Invitrogen). Clones were analyzed and selected, and the protein expression was induced as already described 34, 35. To purify the protein, the culture supernatant was collected after 72 h, centrifuged at 4.300 g for 20 min at 4 °C and filtered on nitrocellulose (pore size 0.22 μm). The filtrate was concentrated about 20 fold using an Amicon® stirred cell equipped with an Ultracel 10 kDa ultrafiltration disc (Amicon Bioseparations, Jaffrey, NH, U.S.A.). The concentrate was dialyzed overnight at 4 °C against 20 mM HEPES, pH 7.4, 300 mM NaCl and then applied to a HisPrep FF (16/10) Ni-Sepharose Fast Flow column (GE Healthcare, Milan, Italy) connected to an AKTA PRIME PLUS system (GE Healthcare). After loading the sample, the column was washed with 20 mM HEPES, pH 7.4, 300 mM NaCl, 20 mM imidazole until the absorption at 280 nm reached the baseline. The bound mouse VC1-His was eluted with a linear gradient of 20 mM HEPES, pH 7.4, 300 mM NaCl, 600 mM imidazole. After analysis of the eluted material by SDS-PAGE, the fractions were pooled and concentrated by using filter units with a 10 kDa cut-off (Amicon Bioseparations, Jaffrey, NH, U.S.A.) and the buffer was exchanged to 0.9% NaCl.

### Isolation and treatment of hcFbs

#### Isolation

HcFbs were isolated from human auricle fragments obtained from female patient that underwent cardiac surgical intervention, following written informed consent and in compliance with the Helsinki Declaration upon approval of the local ethical committee IRCCS IEO and Centro Cardiologico Monzino (protocol CCFM C9/607), or female cadaveric donors of Fondazione Banca dei Tessuti di Treviso (MTA n° 257/A1/2016; Table S1). Cells were isolated and characterized by flow cytometry as described in ref 33. Rat alveolar R3/1 cells expressing FL-RAGE (R3/1-FL-RAGE) or not (R3/1-pLXSN) were used as positive or negative control, respectively 9. An ELISA kit (#DY1145, R&D Systems Inc.) was used for sRAGE detection in the supernatant of hcFbs after seeding cells (2.5x10^5^) in 6 well-plate (Costar, Kennebunk, ME, USA) in starvation medium (IMDM, 10000 U/ml Penicillin, 10000 μg/ml Streptomycin and 20 mmol/l L-Glutamine with 1% FBS) for 48 h.

#### Treatment

HcFbs were used at the 6^th^ passage for all experiments. HcFbs (9×10^4^) were starved at 37 °C o.n. in IMDM supplemented with 1% FBS and treated with human VC1 domain of sRAGE produced in *Pichia pastoris* 34 at 1 or 5 µg/mL or with 10 ng/ml TGF-β1 (#100-21; Peprotech, Rocky Hill, NJ, USA), as positive control, for 4 to 84 h. Signaling experiments were performed treating cell with 5 µg/mL sRAGE, 10 ng/ml TGF-β1 or combination of both for 20 minutes.

For Immunofluorescence, hcFbs (9×10^4^) were incubated with an antibody for Collagen I (1 µg/ml; #ab34710, Abcam), α-SMA (1 µg/ml; #ab5694; Abcam) or Phospho-Smad2-3 (P-Smad2-3; 1:200; #44-244G, Thermo Fisher) overnight (o.n.) at 4 °C. Images were taken using an Apotome microscope (Zeiss) with a 20× objective and quantified with Axiovision Software™ Rel 4.7 (Zeiss); positive cellular area (densitometric units/µm^2^) was determined in 40-50 cells/condition. Experiments were repeated 4 times for three different donors. P-Smad2-3 images were also taken using LSM710 confocal scanning microscope (Carl Zeiss) and 40× objective. Nuclear signal area (densitometric units/µm^2^) of about 20 cells/condition was quantified with Axiovision Software™ Rel 4.7 (Zeiss).

Collagen release in the supernatant of hcFbs was assessed by Sircol kit (#S1000, Biocolor, Carrickfergus, UK) following manufacturer's protocol. Quantification of secreted TGF-β1 in the supernatants of cells was assessed by ELISA assay (#DB100B, R&D Systems) following manufacturer's protocol. Experiments were repeated 4-6 times for three different donors.

Cell proliferation was assessed by Incucyte system (Essen Instruments, Ann Arbor, MI, USA). HcFbs (5×10^3^) were seeded in 96-well plates in starvation medium and incubated o.n. at 37 °C. Medium was replaced with IMDM plus 5% FBS alone or containing sRAGE or TGF-β1 and NucLight Rapid Red Reagent for nuclei labeling (1:1000; #4717, Sartorius, Varedo, Italy). Proliferation was evaluated for 84 h and images were acquired every 2 h. Growth curves were extrapolated considering red nuclei object count/image normalized to h “0”. Slopes were determined with Origin 7.0 (Origin 7, Version 2002; OriginLab Corporation, Northampton, MA, USA) between 20 and 50 h. Treatments were performed in triplicates and results were averaged. The experiment was repeated twice for three different donors.

Cell Cycle distribution was carry out using propidium iodide (PI) incorporation. Briefly, 2.0×10^5^ hcFbs were seeded in 12-well plate and cultured in IMDM supplemented with 5% FBS containing sRAGE or TGF-β1 for 72 h. Cells were washed in PBS, fixed with cold ethanol 96% and stored at 4 °C for 24 h. Then, cells were washed with PBS-FBS 5% and incubated for 2 h in the dark with a solution of 25 μg/mL PI/RNAse (Cat #556746, Calbiochem, San Diego, CA, USA). PI incorporation was measured using BD FACSFGallios (BD Bioscience, SanJose, CA, USA) and the percentage of cells in G0/ G1, S and G2/M phases was determined with the ModFit software (BD Bioscience). At least 5000 events were recorded for each analysis. The experiment was repeated at least three times in two different donors.

TGFbetaR1 and P-Smad2-3 protein expression was determined by flow cytometry. One hundred thousand cells were collected, washed with PBS, fixed and permeabilized with BD Cytofix/Cytoperm™ fixation/permeabilization Kit (#554714, BD Bioscience) and stained with primary antibodies for TGFbetaR1 (1 µg/10^6^ cells, ABF17-I, Sigma-Aldrich) and P-Smad2-3 (1:800, #8828, Cell Signaling Technology) for 30 minutes at rt. After wash, cells were incubated with the fluorochrome-labeled secondary antibody (10 µg/mL, A-11034, Invitrogen) for 30 minutes at rt in dark conditions. Five thousand events were acquired on a BD FACS Gallios (BD Bioscience). Data were analyzed with Kaluza Software (Beckman Coulter, Brea, CA, USA) and reported as percentage of positive cells (mean ± SD). The experiments were repeated at least three times in three different donors.

### Western Blotting

Indicated organs or hcFbs were homogenized in RIPA buffer with proteases (#P8849, Sigma) and phosphatases (#04906837001, Roche, Mannheim, Germany) inhibitors. Membranes were probed with antibodies against RAGE (RAGE N-term, 1 µg/ml; #AF1145, R&D Systems Inc.), Collagen I (1 µg/ml; #ab34710, Abcam), Collagen III (0.5 µg/ml; #ab7778, Abcam), α-SMA (0.2 µg/ml; #ab5694; Abcam), TGFbetaR1 (1 µg/ml, ABF17-I, EMD Millipore, USA), βTubulin (1 µg/ml; #T-6199, Sigma-Aldrich), βactin (1:1000; #A5441, Sigma) or GAPDH (0.2 µg/ml, sc-25778, Santa Cruz Biotechnology, Dallas, Texas, USA). For signaling experiments, total Smad2-3 (1:1000, #8685, Cell Signaling Technology) and P-Smad2-3 (1:1000, #8828, Cell Signaling Technology) were used according to manufacturer's instructions. Red Ponceau (P7170, SIGMA) was used as normalizer of protein content. Proteins were visualized by Clarity^TM^ or Clarity Max ^TM^ Western ECL substrate (#170-5060, 170-5062, Biorad) and acquired with a ChemiDoc™ MP Imaging System (Biorad, Hercules, CA, USA). Protein bands were quantified by densitometry analysis using ImageJ (rsb.info.nih.gov/ij).

### RT-qPCR

LV or lungs were homogenized with the TissueLyser (Qiagen, Hilden, Germany) and RNA extracted by RNeasy Mini Kit (Qiagen). RNA was treated with the TURBO DNA-free Kit (Invitrogen). RNA from hcFbs was extracted using Illustra RNAspin Mini kit (#25-0500-72; GE Healthcare). Experiments were repeated four times on three different donors. RT-qPCR was performed on a Bio-Rad iCycler Thermal Cycler (Hercules, CA, USA) with iQ5 Multicolor Real-Time PCR Detection System using the iQ SYBR Green Supermix (Bio-Rad) and specific oligos (Table S2). The relative gene expression was determined using the 2-ΔΔCT method and was normalized to the average of 4 house-keeping genes (*Ppih*, *Hprt*, *Gusb*, *Ldha*) for murine tissues or *HPRT* gene for hcFbs.

### Statistical analysis

Data were analyzed with GraphPad Prism 7 (GraphPad Software, Inc, La Jolla, CA, USA) or SAS 9.4 programs. D'Agostino or Shapiro-Wilk test was used for the distribution of parameters. Student's t-test was used for comparison between two groups. Analysis between more than two groups was conducted by 1- or 2-way ANOVA with Bonferroni post-hoc test, when appropriate, and indicated in the figure legend. The crude relation of sRAGE and echocardiographic parameters was evaluated by Pearson linear correlation. The independent effect of sRAGE on echocardiographic parameters was evaluated by multivariable linear regression after adjusting for age. Skewed distributed variables were transformed to base 10 logarithm. Data were presented as mean ± SD. P<0.05 was considered significant.

## Results

### RAGE deficiency accelerates age-dependent LV dimension changes without affecting LV function

To investigate RAGE involvement in cardiac aging, we generated three groups of *Rage-/-* and WT female mice of different ages: 2.5- (Young), 12- (Middle-age; MA) and 21-months-old (Old). Body weight (BW) increased in MA mice of both genotypes relative to Young animals; Old *Rage-/-* mice exhibited a further and significant rise in BW compared to the corresponding WT group (Table 1). LV mass increased with age in animals of both genotypes but was significantly higher in MA and Old groups of *Rage-/-* mice compared to age-matched WT mice (Table 1). LV-to-tibia length ratio (LV/TL) was higher in MA and Old groups of both genotypes relative to Young animals and tended to further increase in *Rage-/-* animals (Table 1).

**Figure 1:**
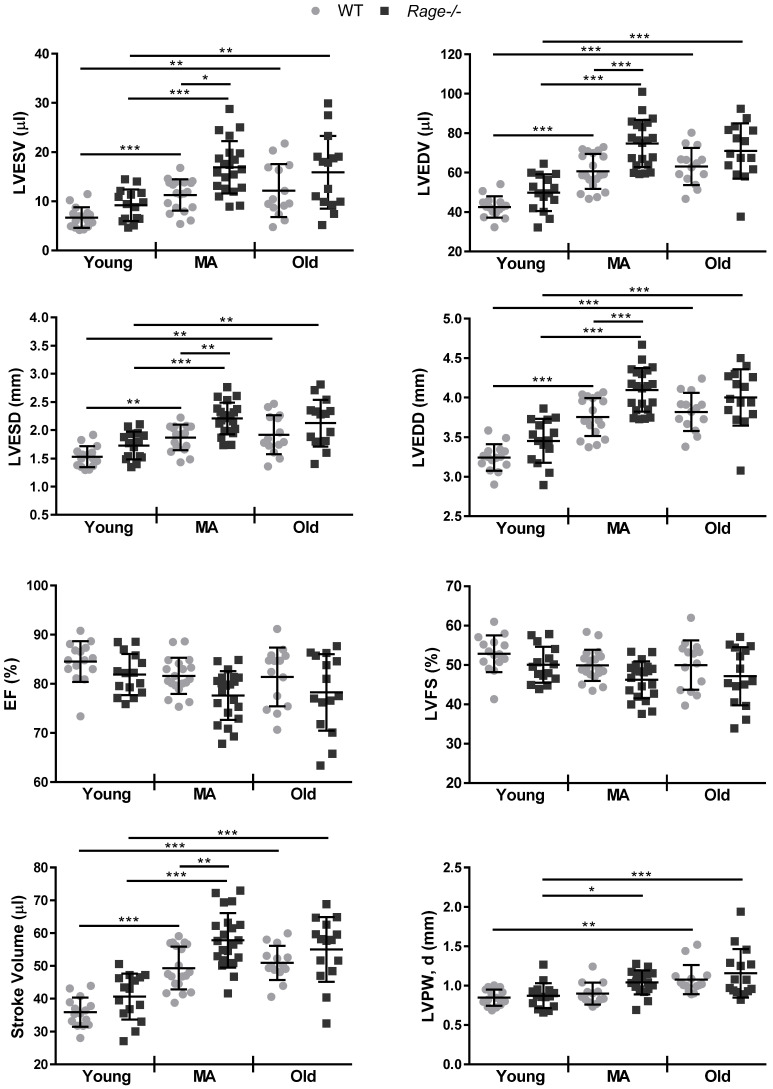
** RAGE deficiency accelerates age-dependent LV dimension changes without affecting LV function.** Echocardiography was performed on WT and *Rage-/-* mice aged of 2.5 (Young), 12 (Middle-age, MA) and 21 months (Old) to measure Left Ventricle (LV) end-diastolic (LVEDV) and end-systolic (LVESV) volumes, LV end-diastolic (LVEDD) and end-systolic (LVESD) diameters, LV ejection fraction (LVEF), LV fractional shortening (LVFS), Stroke Volume, and LV posterior wall in diastole (LVPW, d). Young WT n=15, Young *Rage-/-* n=15, MA WT n=18, MA *Rage-/-* n=21, Old WT n=14, Old *Rage-/-* n=15. Each dot represents a mouse; mean ± SD are shown (*, P<0.05; **, P<0.01; ***, P<0.001; 2-way ANOVA plus Bonferroni post-hoc test for multiple comparisons).

Then, we assessed LV dimensions and function. WT mice showed a significant increase in LV volumes and diameters in systole and diastole (LVESV, LVESD, LVEDV, LVEDD) and stroke volume (SV) in the MA group compared to Young group, and no additional increase in the Old group (Figure 1). Notably, *Rage-/-* mice displayed higher LV dimensions and SV in comparison to age-matched WT mice, reaching a significant difference in the MA group (Figure 1). Of note and opposite to WT mice, Old *Rage-/-* animals had tendency to lower LV dimensions compared to the *Rage-/-* MA group (Figure 1). LV ejection fraction (LVEF) and fractional shortening (LVFS) remained similar among all groups of animals of both genotypes with a tendency to decrease with age and in a more marked way in *Rage-/-* mice (Figure 1). Finally, besides an age-associated increase in LV posterior wall in diastole (LVPW, d), in particular among Young and Old animals, we did not observe significant differences between WT and *Rage-/-* mice across all the age groups (Figure 1).

Thus, RAGE deficiency accelerates age-induced LV dimension changes without influencing systolic function.

### *Rage-/-* mice exhibit enhanced age-associated cardiac fibrosis and early signs of HF

**Figure 2:**
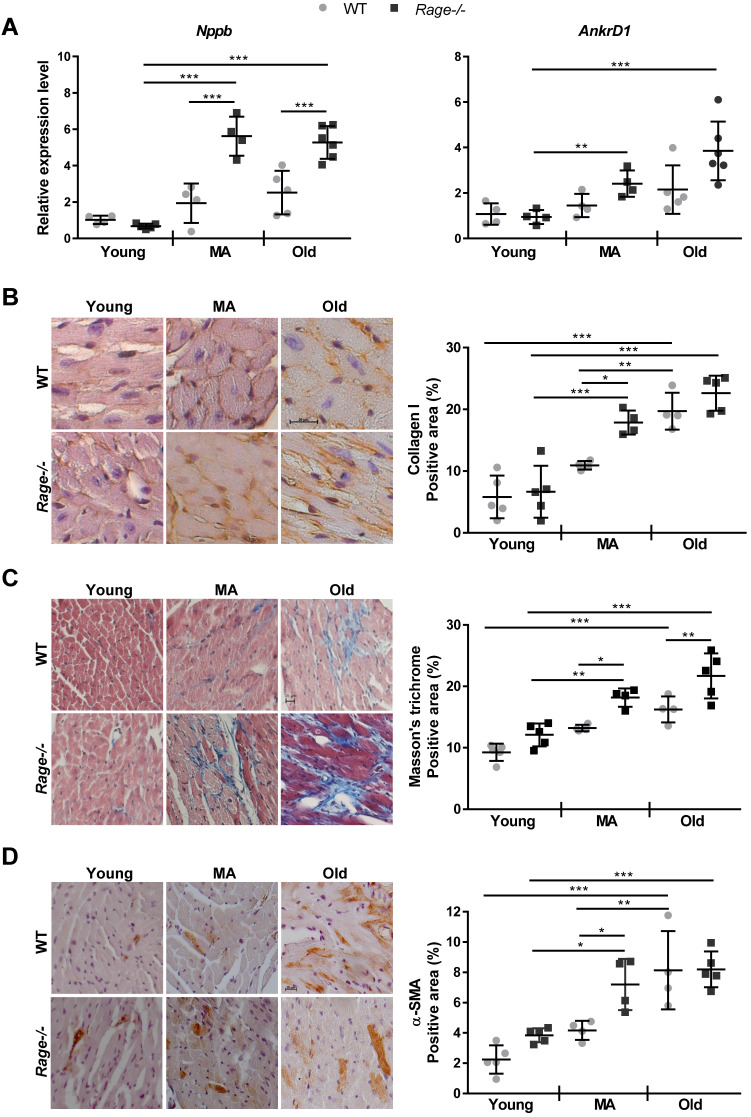
** RAGE deficiency promotes the expression of HF marker genes and accelerates age-associated cardiac fibrosis. (A)** Relative expression of BNP (*Nppb*) and ANKRD1 (*Ankrd1*) genes. Young WT n=4, Young *Rage-/-* n=4-5, Middle-age (MA) WT n=4, MA *Rage-/-* n=4, Old WT n=5, Old *Rage-/-* n=6. **(B)** (Left panel) Representative images of LV sections stained with an antibody against Collagen I. Bar, 20 µm. (Right panel) Quantification of Collagen I signal. Young WT n=5, Young Rage-/- n=5, MA WT n=4, MA Rage-/- n=4, Old WT n=4, Old Rage-/- n=5. **(C)** (Left panel) Representative images of LV sections stained with Masson's trichrome. Bar, 20 µm. (Right panel) Quantification of Masson's trichrome signal. Young WT n=5, Young *Rage-/-* n=5, MA WT n=4, MA *Rage-/-* n=4, Old WT n=4, Old *Rage-/-* n=5. **(D)** (Left panel) Representative images of LV sections stained with an antibody anti-α-SMA. Bar, 20 µm. (Right panel) Quantification of α-SMA signal. Young WT n=5, Young *Rage-/-* n=5, MA WT n=4, MA *Rage-/-* n=4, Old WT n=4, Old *Rage-/-* n=5. Each dot represents a mouse; mean ± SD are shown (*, P<0.05; **, P<0.01; ***, P<0.001; 2-way ANOVA plus Bonferroni post-hoc test for multiple comparisons).

In the light of above data, we evaluated the expression of HF marker genes such as *Nppb* and *AnkrD1,* encoding for Brain Natriuretic Peptide (BNP) and ankyrin repeat domain 1 (ANKRD1; 36), respectively. The Old WT group showed a tendency to increased levels of* Nppb* relative to the Young WT group; interestingly, *Nppb* expression reached the highest level in the MA *Rage-/-* mice compared to the Young *Rage-/-* mice and scored significantly different in MA and Old *Rage-/-* groups compared to age-matched WT animals (Figure 2A). *AnkrD1* expression progressively increased with age in *Rage-/-* but not in WT mice (Figure 2A).

Next, we performed histological analysis to identify the morphological alterations responsible for the age-related LV phenotype of* Rage-/-* mice, considering CM hypertrophy and cardiac fibrosis, which normally increase with aging. First, we evaluated the size of CM on heart sections and observed a similar age-dependent augmentation of CM area in WT and *Rage-/-* animals (Figure S1A). Accordingly, measures of area and major and minor diameters of CM isolated from the hearts of MA *Rage-/-* and WT mice confirmed comparable CM dimensions (Figure S1B-E).

Then, we evaluated cardiac fibrosis by Masson's thricrome staining and in terms of Collagen I deposition. WT animals exhibited a progressive increase of fibrosis with age, with the WT Old group showing the highest Masson's thricrome positivity and Collagen I content in relation to Young WT mice (Figure 2B-C). Of note, *Rage-/-* animals showed a robust accumulation of Collagen I already in the MA group, which scored markedly higher compared to the WT counterpart (Fig. 2B-C). An additional increase in fibrosis was still observed in the Old *Rage-/-* animals (Figure 2C). In order to check whether exaggerated fibrosis observed in the MA *Rage-/-* mice may depend on the enhanced activity of CFs, we counted interstitial α-SMA+ cells. We found that while in WT mice α-SMA+ cells significantly accumulated in the Old group, *Rage-/-* mice showed a substantial presence of α-SMA+ cells already in the MA group, which scored significantly higher compared to its age-matched WT counterpart (Figure 2D; Figure S2A). α-SMA+ cells further increased in the Old *Rage-/-* mice (Figure 2D).

**Figure 3:**
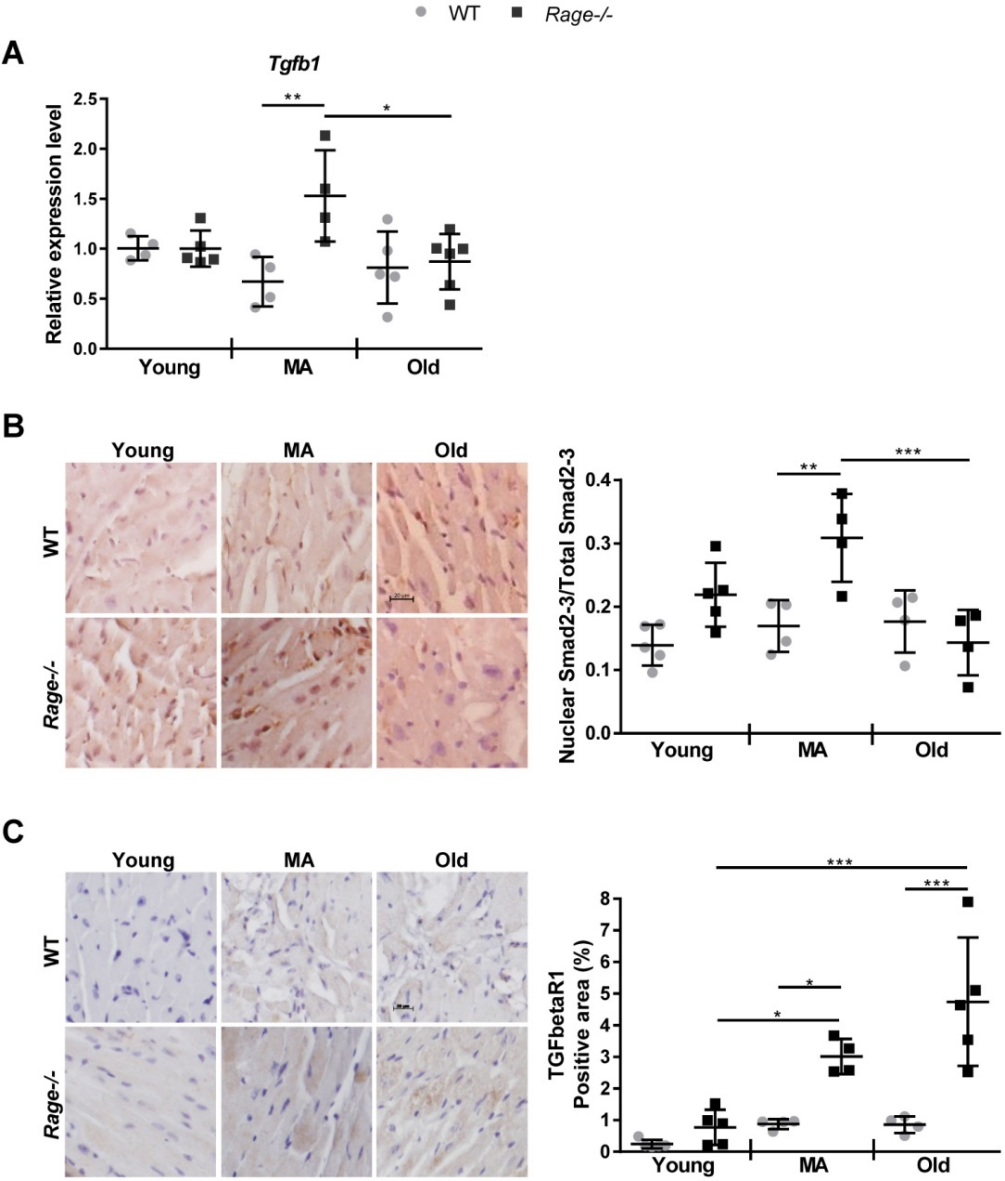
** RAGE deficiency induces age-associated TGF-β signaling. (A)** Relative expression of *Tgfb1*gene. Young WT n=4, Young *Rage-/-* n=4-5, Middle-age (MA) WT n=4, MA *Rage-/-* n=4, Old WT n=5, Old *Rage-/-* n=6. **(B)** (Left panel) Representative images of LV sections stained with an anti-Smad2-3 antibody. Bar, 20 µm. (Right panel) Quantification of Smad2/3 signal. Young WT n=5, Young *Rage-/-* n=5, MA WT n=4, MA *Rage-/-* n=4, Old WT n=4, Old *Rage-/-* n=5. **(C)** (Left panel) Representative images of LV sections stained with an anti-TFGbetaR1 antibody. Bar, 20 µm. (Right panel) Quantification of TFGbetaR1 signal. Young WT n=5, Young *Rage-/-* n=5, MA WT n=4, MA *Rage-/-* n=4, Old WT n=4, Old *Rage-/-* n=5. Each dot represents a mouse; mean ± SD are shown (*, P<0.05; **, P<0.01; ***, P<0.001; 2-way ANOVA plus Bonferroni post-hoc test for multiple comparisons).

Since TGF-β1 is a driver of fibrosis, we assessed its RNA expression in the LV. *Tgfβ1* levels increased in the MA *Rage-/-* animals and were markedly enhanced with respect to age-matched WT mice (Figure 3A). Then, we evaluated the activation of TGF-β1-dependent Smad2/3 proteins and TGFbetaR1 expression 37. Nuclear/activated Smad2-3 was significantly increased in MA *Rage-/-* mice in comparison to the age-matched animals (Figure 3B; Figure S2B), with a pattern of expression similar to *Tgfβ1* across all the groups (Figure 3A-B). Interestingly, TGFbetaR1 expression was slightly enhanced with age in WT animals; however, MA and Old *Rage-/-* mice showed a more marked increase with age that was significantly different from the corresponding WT animals (Figure 3C; Figure S2C).

Finally, we evaluated the presence of CD45+ cells in LV sections. The number of infiltrating cells gradually increased in the MA and Old WT groups compared to WT Young animals, while it remained unchanged in *Rage-/-* mice across the age groups (Figure S3).

These data demonstrate that RAGE deficiency favors the onset of an age-dependent HF phenotype, mostly by accelerating fibrosis through the activation of the TGF-β1/TGFbetaR1/Smad2-3 pathway, whereas it does not affect CM hypertrophy and tissue infiltration by inflammatory cells.

### RAGE expression in LV during aging

**Figure 4:**
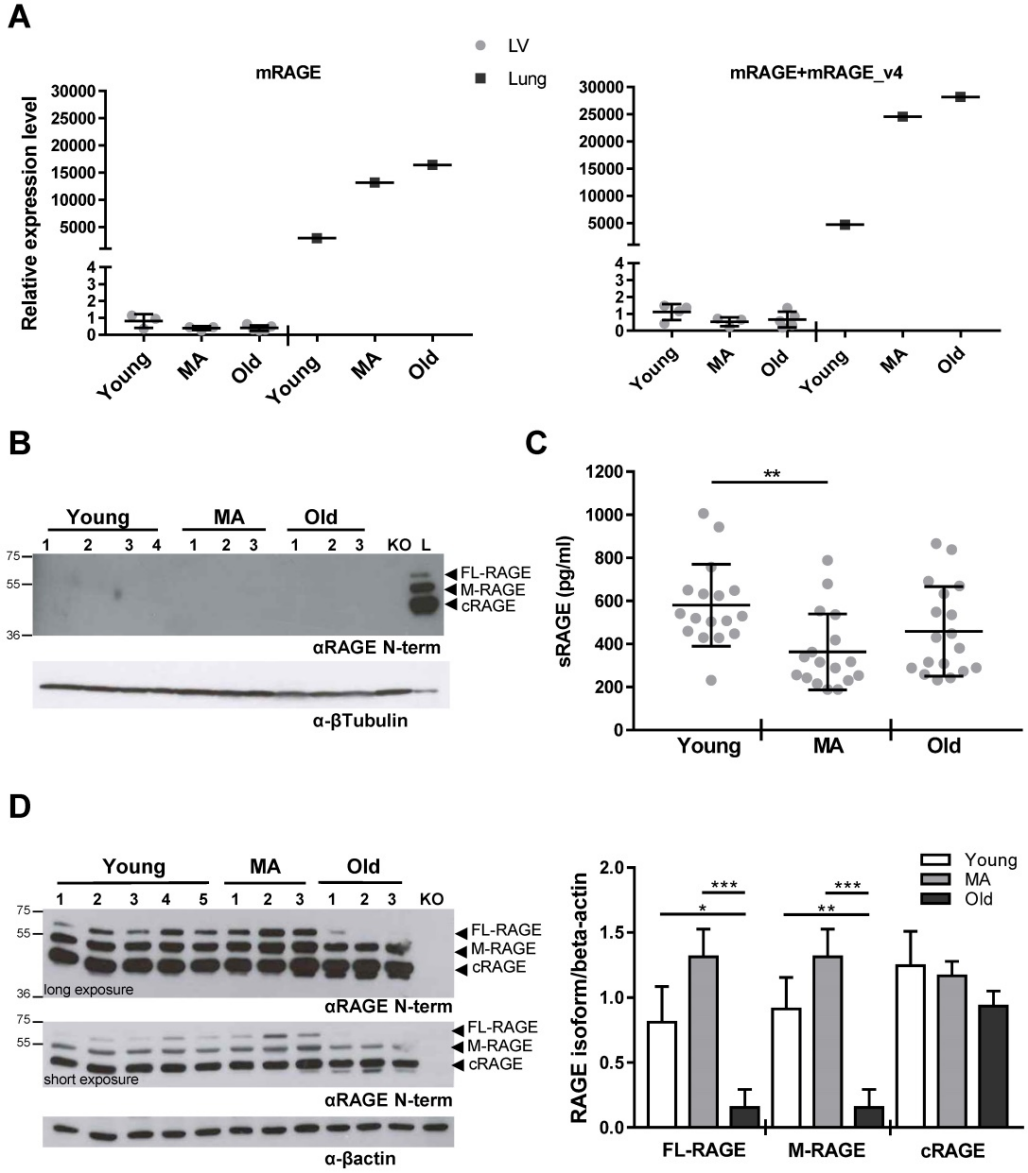
**Circulating sRAGE decreases with age in mice. (A, B)** RAGE isoforms are not detectable in LV tissue. (A) mRNA levels of mRAGE or mRAGE+mRAGE_v4 isoforms in LV or lung (positive control) of indicated WT age groups. (B) Forty µg of LV protein lysate of the indicated age groups of WT mice were probed with an antibody α-RAGE (RAGE N-term). The same amount of LV from a Young *Rage-/-* mouse (KO) was used as negative control, and 10 µg of lung protein lysate of a WT mouse (L) as positive control for RAGE expression. β-tubulin was used as loading control (n=3-4 per group). **(C)** Quantification of sRAGE in the serum of the indicated age groups of WT mice; Young n=17, MA n=17 and Old n=18. **(D)** (Left panel) Ten µg of lung protein lysate of the indicated age groups of WT mice were probed with an antibody against RAGE (RAGE N-term) and a long and a short exposure are shown. Ten µg of lung protein lysate from a Young *Rage-/-* mouse (KO) was used as negative control. α-actin was used as loading control. (Right panel) Quantification of RAGE isoforms in the lung protein lysate of the indicated age groups of WT mice (n=3-5 per group). Each dot represents a mouse; mean ± SD are shown (*, P<0.05; **, P<0.01; ***, P<0.001; 1-way ANOVA plus Bonferroni post-hoc test for multiple comparisons).

We have published that embryonic and adult murine lungs express three RAGE variants, FL-RAGE, M-RAGE and the soluble cleaved cRAGE 15. In order to examine possible alterations in the expression of RAGE mRNA isoforms during intrinsic cardiac aging, we determined the expression of mRAGE and mRAGE_v4 transcripts encoding for FL-RAGE and M-RAGE, respectively, in LV of Young, MA and Old WT mice. Taking advantage of the lack of exon 9 in mRAGE_v4, we used two pairs of primers, one that amplifies only mRAGE and the second that amplifies both mRAGE+mRAGE_v4 (Table S2). Figure 4A shows that RAGE mRNA was extremely low in the LV as compared to lung and no variations were observed during aging. Accordingly, RAGE protein was not found in the LV of WT animals in any age group, while protein bands corresponding to FL-RAGE, M-RAGE and c-RAGE were clearly visible in the lung lysate (Figure 4B).

Our results show that there is an extremely low, if any, expression of all RAGE isoforms in the LV and their expression does not change with aging.

### Circulating sRAGE decreases with aging and inversely associates with LV diastolic dimensions

Circulating sRAGE decreases with age in healthy patients 18. Thus, we assessed the concentration of sRAGE in the serum of all WT mouse age groups. sRAGE averaged about 600 pg/ml in Young animals, markedly dropped in the MA group and was not significantly different in the Old group compared to the MA group (Figure 4C).

To determine whether circulating sRAGE variations may reflect LV phenotype changes during aging, we performed a Pearson correlation analysis between serum levels of sRAGE and echocardiographic parameters, combining the data of all age groups of WT animals. A significant inverse correlation was found among LVEDV, LVEDD, SV and sRAGE (Table 2). To explore the age-independent association of sRAGE and echocardiographic parameters, we performed a linear multivariate regression adjusting for age and found that LVEDV, LVEDD, and SV still associated with sRAGE levels (Table 2).

Since the lung is the organ that highly expresses all RAGE isoforms 38, we checked whether the changing in circulating sRAGE, which is mainly constituted by cRAGE in mice 38, could depend on variation in pulmonary RAGE expression. Western blot revealed that FL-RAGE and M-RAGE isoforms had the tendency to be higher in MA mice compared to Young ones while decreased significantly in the Old group in relation to MA and Young animals (Figure 4D). cRAGE did not vary significantly with age (Figure 4D). Then, we determined RAGE isoforms expression in other organs such as brain, liver, spleen, and muscle without detecting any specific signal (Figure S4).

Our observations indicate that circulating sRAGE decreases with age and associate with LV diastolic dimensions in mice. Moreover, the data also suggest that in mice the circulating sRAGE may derive from the lung.

### sRAGE reduces pro-fibrotic activities of hcFbs through inhibition of TGFbetaR1/Smad2-3 activation

We hypothesized that circulating sRAGE has a direct role in preventing cardiac fibrosis. Thus, we tested the effect of recombinant sRAGE on the activity of hcFbs of mesenchymal origin and their differentiation into MyoFbs. We confirmed no detectable protein expression of FL-RAGE or released sRAGE, respectively (Figure S5; 33), in hcFbs isolated from three different female donors (Table S1).

**Figure 5:**
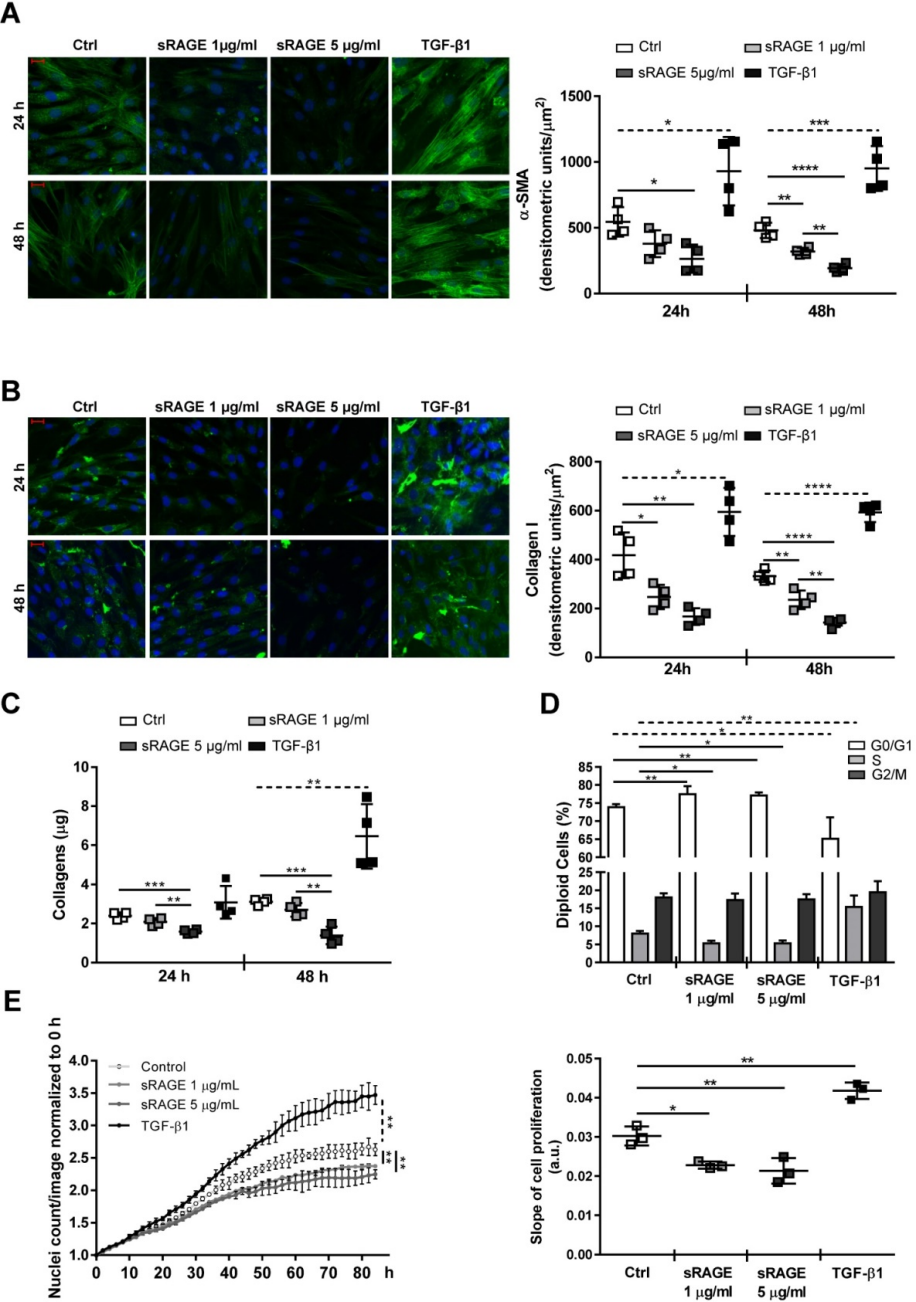
**Recombinant sRAGE reduces pro-fibrotic activity and proliferation of hcFbs. (A-C)** HcFbs were stimulated with vehicle (Ctrl) or the indicated concentrations of sRAGE for 24 or 48 h. TGF-β1 (10 ng/ml) was used as positive control. **(A)** (Left panel) Representative images of α-SMA protein expression (green). Nuclei were stained with Hoechst (blue). Bar, 20 μm. (Right panel) Quantification of α-SMA expression. **(B)** (Left panel) Representative images of Collagen I expression (green). Nuclei were stained with Hoechst (blue). Bar, 20 μm. (Right panel) Quantification of Collagen I expression. **(C)** Collagens released in hcFbs supernatant. **(D)** Cell cycle distribution of hcFbs stimulated for 72 h with the indicated concentration of sRAGE or TGF-β1 (10 ng/ml). **(E)** (Left panel) Proliferation of hcFbs treated with indicated concentrations of sRAGE and TGF-β1 (10 ng/ml) recorded every 2 h; statistical differences were calculated 84 h after treatment. (Right panel) Slope of cell proliferation from 20 to 50 h. Each dot represents a biological replicate; mean ± SD are shown (*, P<0.05; **, P<0.01; ***, P<0.001; ****, P<0.0001; 1-way ANOVA plus Bonferroni post-hoc test for multiple comparisons between Ctrl and sRAGE at the same time point; dotted line: t-test between Ctrl and TGF-β1; n=3-4).

HcFbs were stimulated for 24 and/or 48 h with recombinant human RAGE VC1 domains (1 or 5 µg/ml sRAGE) or TGF-β1 (10 ng/ml). Accordingly, cellular expression of α-SMA and Collagen I proteins (Figure 5A-B; Figure S6) and the release of Collagen species in the cell supernatants (Figure 5C) were dose-dependently reduced by sRAGE, while TGF-β1 had opposite effect. Besides, contrary to TGF-β1, sRAGE also inhibited hcFbs proliferation by increasing the number of cells in G0/G1 and reducing those in S phase (Figure 5D-E). Similar results were obtained in cells isolated from three female donors.

**Figure 6:**
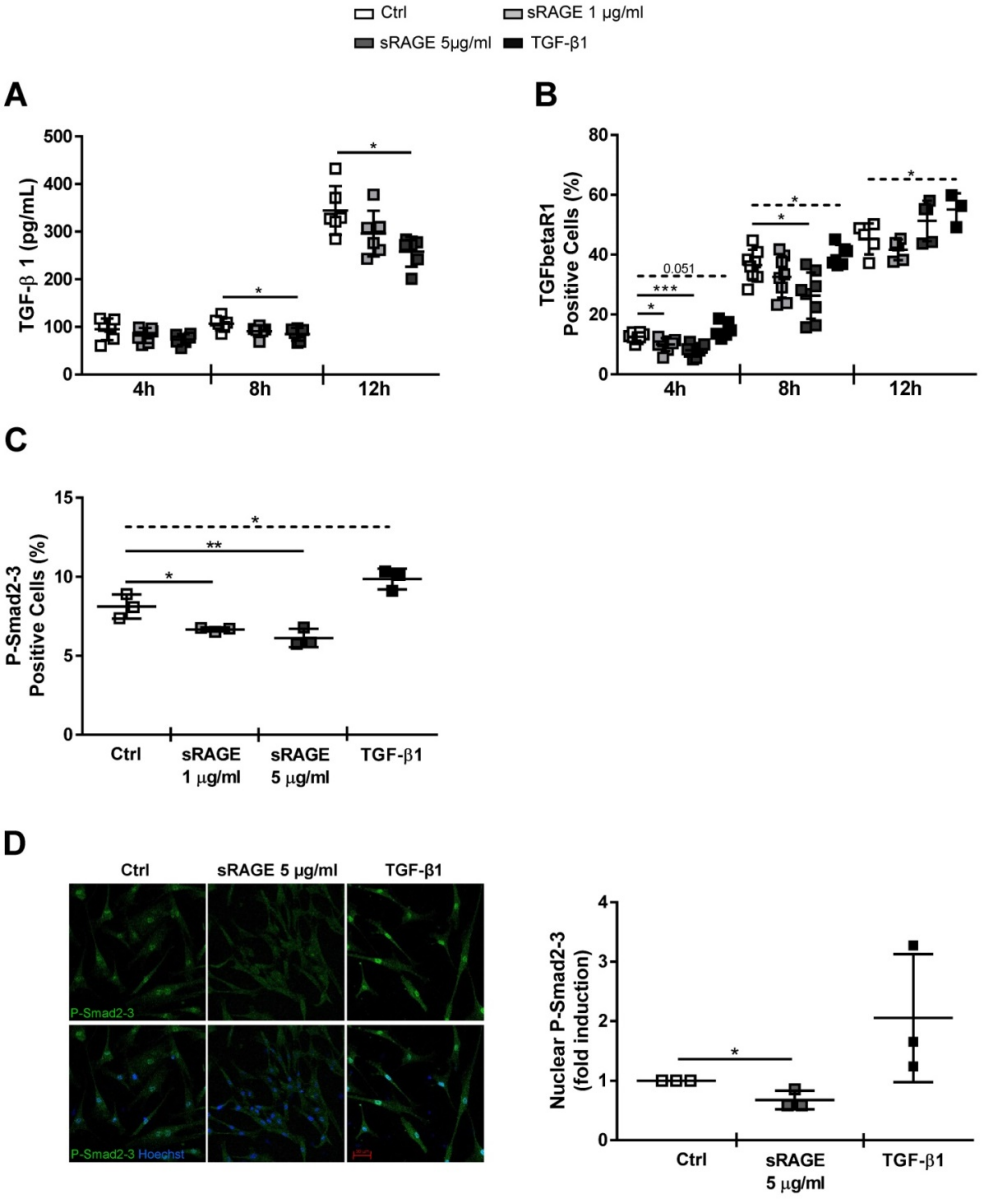
**Recombinant sRAGE reduces TGFbetaR1 expression and inhibits Smad2/3 signaling in hcFbs.** HcFbs were stimulated with nothing (Ctrl) or the indicated concentrations of sRAGE for 4, 8 or 12 h. TGF-β1 (10 ng/ml) was used as positive control when indicated. **(A)** TGF-β1 released in the supernatant of cells determined by ELISA; n=6. **(B)** TFGbetaR1 expression determined by FACS analysis; n=3-8. **(C)** Quantification of P-Smad2-3 expression determined by FACS analysis 4 h after stimulation; n=3. **(D)** (Left panel) Representative immunofluorescence images for P-Smad2-3 (green) 4 h after stimulation; nuclei were stained with Hoechst (blue). Bar, 50 μm. (Right panel) Quantification of nuclear P-Smad2-3 expression; n=3 Each dot represents a biological replicate; mean ± SD are shown (*, P<0.05; **, P<0.01; ***, P<0.001; t-test or 1-way ANOVA plus Bonferroni post-hoc test for multiple comparisons between Ctrl and sRAGE at the same time point; dotted line: t-test between Ctrl and TGF-β1).

Next, we investigated whether sRAGE interferes with differentiation of hcFbs into MyoFbs by influencing the activation of TGF-β1 pathway. sRAGE was not able to directly counteract the basal and TGF-β1-dependent Smad2-3 activation (Figure S7). Then, we assessed whether sRAGE could influence the basal release of TGF-β1. Figure 6A shows that TGF-β1 level was dose-dependently reduced by 8 h of sRAGE treatment; a more substantial increase of TGF-β1 secretion (around 300 pg/ml) was observed at 12 h. Interestingly, TGFbetaR1 protein expression was significantly downregulated by sRAGE already after 4 h of treatment and the effect was maintained at 8 h and lost 12 h later (Figure 6B). Accordingly, basal activation of P-Smad2-3 was clearly reduced 4 h after administration of sRAGE (Figure 6C-D). As control, TGF-β1 was able to induce both phosphorylation of Smad2-3 and TGFbetaR1 expression (Figure 6B-D).

Together, these data demonstrate that sRAGE has a direct capability to modulate hcFbs functions and inhibits their differentiation into MyoFbs. Mechanistically, sRAGE induces a reduction of TGFbetaR1 expression and subsequent inhibition of P-Smad2-3 activation that, in turn, reduces the basal release of TGF-β1 and production of collagens.

### Administration of sRAGE reduces age-associated cardiac fibrosis *in vivo*

**Figure 7:**
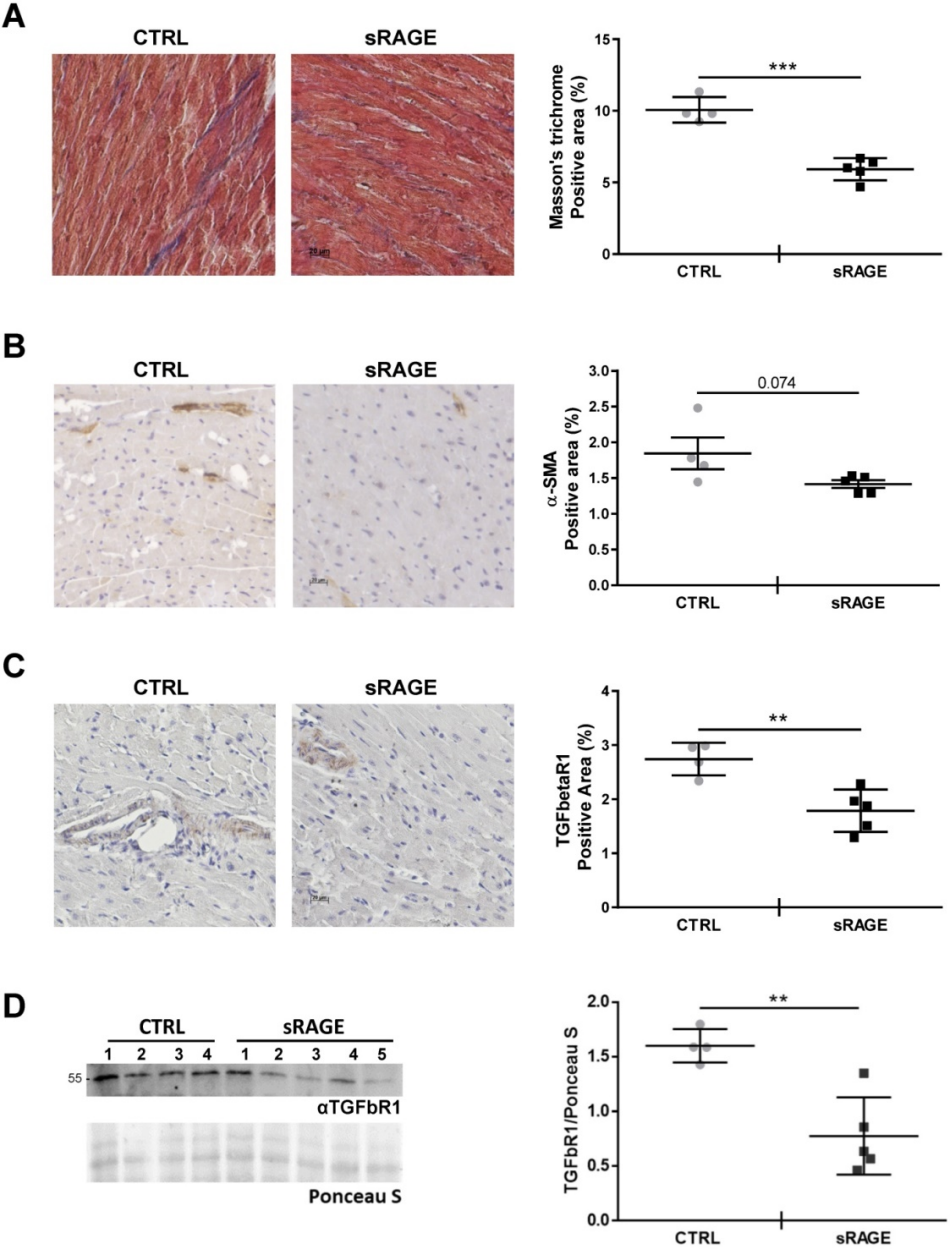
**Recombinant sRAGE reduces fibrosis and TGFbetaR1 expression *in vivo*.** MA WT mice were injected daily with about 22 μg of recombinant murine sRAGE (sRAGE) for 8 days or equal volume of physiological salt solution (CTRL). **(A)** (Left panel) Representative images of LV sections stained with Masson's trichrome. Bar, 20 µm. (Right panel) Quantification of Masson's trichrome signal. CTRL n=4, sRAGE n=5. **(B)** (Left panel) Representative images of LV sections stained with an antibody anti-α-SMA. Bar, 20 µm. (Right panel) Quantification of interstitial α-SMA signal. **(C)** (Left panel) Representative images of LV sections stained with an anti-TFGbetaR1 antibody. Bar, 20 µm. (Right panel) Quantification of TFGbetaR1 signal. **(D)** (Left panel) Western blot analysis of 35 µg of LV protein lysate probed with an antibody αTGFbR1. Red Ponceau staining (Ponceau S) was used as loading control. (Right panel) Quantification of TGFbR1 expression in the LV protein lysate. CTRL n=4, sRAGE n=5. Each dot represents a mouse; mean ± SD are shown (**, P<0.01; ***, P<0.001; t test).

In order to assess that levels of circulating sRAGE regulate the age-dependent cardiac fibrosis *in vivo*, we injected recombinant murine sRAGE in MA WT mice daily for eight consecutive days. Animals treated with sRAGE exhibited a significant lower amount of collagen fibers and fibrosis (Figure 7A; Figure S8) and showed the tendency to a lower percentage of interstitial α-SMA+ cells compared to control mice (CTRL; Figure 7B). Furthermore, sRAGE reduced the expression of TGFbetaR1 in the cardiac tissue (Figure 7C-D). We did not observe relevant changes in LV diameters and volumes determined with echocardiographic analysis (Table S3) likely because the timing of sRAGE administration is too short to affect cardiac dimensions.

Hence, our data demonstrate that circulating sRAGE acts as cardio-protective molecule with anti-fibrotic activity *in vivo*.

## Discussion

Here, we investigated the role of RAGE in myocardial aging and identified sRAGE as a regulator of cardiac fibroblasts activity and cardiac fibrosis *in vivo*. In mice, cardiac aging is characterized by LV mass growth and a consequent increase in wall thickness and LV dimensions with preserved EF. Cardiac hypertrophy and interstitial fibrosis are compensatory mechanisms that sustain age-dependent LV remodeling and counteract CM loss 2, 3. We found that *Rage-/-* mice exhibit an exacerbation of LV end-diastolic and -systolic volumes and diameters while aging when compared to WT mice. Of note, the major differences with aging and between the two genotypes occurred during the transition from Young to MA (Figure 1). In addition, *Rage-/-* mice experienced a slight reduction in both EF and FS relative to age-matched WT mice, indicating that *Rage-/-* mice were still able to compensate for the acceleration of LV remodeling in response to aging. This is also suggested by the increase in the SV of *Rage-/-* mice (Figure 1). Nevertheless, MA and Old *Rage-/-* animals showed signs of HF onset, since they express higher levels of HF marker genes *Nppb* and *AnkrD1* (Figure 2A). Interestingly, RAGE deletion does not influence the physiological increase of CMs hypertrophy and LV wall thickness, which were comparable to WT animals across all the age groups (Figure S1), but clearly affected myocardial fibrosis (Figure 2B-D) suggesting that exacerbation of age-dependent LV remodeling may be due to an altered activity of CFs. Indeed, *Rage-/-* MA and Old mice show enhanced collagen fibers deposition and a higher number of α-SMA+ MyoFbs (Figure 2B-D; Figure S2A), probably due to the upregulation of the fibrotic gene *Tgfb1* (Figure 3A), which is crucial in promoting the trans-differentiation of CFs into MyoFbs and synthesis of collagens 1 through the activation of TGFbetaR1/Smad2-3 37 (Figure 3B-C; Figure S2B-C). Hence, *Rage-/-* mice showed an exacerbation of the myocardial remodeling, in particular of fibrosis, compared to WT animals while aging.

Cardiac aging features also low-grade chronic tissue inflammation and senescent murine hearts accumulate pro-inflammatory M1 macrophages that contribute to cardiac impairment 39, 40. We observed that CD45+ cells tended to increase in the LV of Old WT but not MA WT, nor in *Rage-/-* mice (Figure S3), indicating that the LV changes in the MA WT group may be independent of low-grade inflammation, which instead may play a role during the transition to the Old stage.

Because *Rage-/-* mice lack both FL-RAGE and sRAGE, which have opposite functions, the phenotype of these animals results from the absence of both proteins in relation to the type of injury (acute *vs* chronic), level of inflammation and concentration of ligands. RAGE is known to induce sustained inflammation associated to cancer 22 and CVDs such as atherosclerosis, MI and myocarditis 10, 13, 23, 41. In these pathological contexts, *Rage-/-* mice are characterized by decreased recruitment of inflammatory cells to the damaged tissue and local inflammation. This is due to FL-RAGE interaction with a plethora of pro-inflammatory and chemoattractant ligands like HMGB1, Mac-1, adhesion and extracellular matrix molecules that mediate trans-migration of inflammatory cells to the site of injury and cell activation 9, 11, 12, 42. On the other hand, sRAGE exerted a protective action in several experimental models of chronic diseases 19-23. Of note, different groups have recently published that *Rage-/-* mice develop lung fibrosis with age 30, 31, highlighting that these animals progress to a pathological phenotype. Kumar et al. showed that the lungs of *Rage-/-* mice have a distorted alveoli pattern and decreased pulmonary function resulting from impaired DNA repair 31. With age, this leads to an accumulation of fibrosis and ultimately cancer 30, 31. The same authors observed the presence of fibrosis also in the brain, kidney and heart 31. Our study confirms and extends these findings by showing for the first time that, in the heart, chronic deletion of *Rage* gene exacerbates age-dependent myocardial changes by enhancing the progression of interstitial fibrosis. A gene expression profiling of these mice would be helpful to explore all mechanisms responsible for their age-dependent cardiac phenotype.

Notably, our findings demonstrate that in WT mice circulating concentration of sRAGE regulates the age-associated myocardial remodeling by exerting an anti-fibrotic function. Indeed, we observed that: 1. Serum levels of sRAGE decline with age and inversely correlate and associate with LV diastolic dimensions (Figure 4C, Table 2); 2. Administration of sRAGE in MA WT animals reduces cardiac collagens content, the presence of α-SMA+ MyoFbs and TGFbetaR1 expression (Figure 7; Figure S8); 3. The effect of sRAGE on hcFbs activity *in vitro* is consistent with the *in vivo* data. Indeed, sRAGE limites hcFbs proliferation and differentiation into MyoFbs, reduces the production of collagens and TGFbetaR1 expression and Smad2-3 activation (Figure 5-6; Figure S6). Of note, most significant variations in both circulating sRAGE and LV changes, take place during the transition from the Young to the MA group (Figure 1 and 4D). Therefore, the age-dependent decrease of circulating levels of sRAGE in WT mice may regulate the physiological increase of interstitial fibrosis by favoring the transition of cFbs into MyoFbs and the partial and short time restoration of sRAGE levels exers a therapeutic beneficial effect (Figure 7).

sRAGE has scavenger activity and some RAGE ligands such as AGEs are known to accumulate with age, thereby activating inflammation and fibrosis 18, 43, 44. It is also known that sRAGE has a ligand-independent chemotactic function through direct interaction with Mac-1 45. Moreover, RAGE ligands promote cardiac fibrosis inducing activation, migration and proliferation of CFs in a FL-RAGE-independent manner *via* Toll-like Receptors (TLRs) and Peroxisome-Proliferator-Activated Receptor-γ (PPAR-γ) or the Angiotensin II (Ang II) pathway 43, 46, 47. Herein, we demonstrate that sRAGE possesses an autonomous intrinsic anti-fibrotic activity since it is able to inhibit TGFbetaR1 expression and Smad2-3 activation; this in turn reduces the release on endogenous TGF-β1 and eventually collagens production (Figure 6). Hence, the progressive decline of sRAGE with age could systemically both reduce physiological cardio-protection and allow RAGE ligands to exert their fibrotic action through other receptors. The mechanisms by which sRAGE promotes the downregulation of TGFbetaR1 expression may be several, including the induction of microRNAs that have been already shown to target TGFbetaR1 mRNA and control the fibrotic process 48, 49. Future experiments are needed to clarify this aspect.

Aging is characterized by a time-dependent loss of tissue function and change in anatomic structure, which are responsible for diminished resistance to stress 50. The identification of biomarkers able to monitor age-related alterations could help to measure the "biological age" and predict the onset of age-associated pathologies. Epidemiological studies associate lower levels of sRAGE with different CV risk factors 24-26. We have recently published that in a healthy population circulating sRAGE progressively declines and inversely correlates with age 18, pinpointing this soluble receptor as an indicator of aging. Here, we showed that circulating sRAGE decreased with age in mice as well (Figure 4C) and inversely correlated with LV end-diastolic dimensional changes, suggesting it as a possible biomarker of cardiac aging (Table 2). The strength of our findings is that we followed cardiac changes, sRAGE levels and echocardiographic parameters over multiple age groups and in the absence of CVD, as mice do not develop spontaneously hypertension or atherosclerosis 1, 3. Limitations of the present work are the involvement of only female animals and the lack of relation between sRAGE and markers of CVD/hearth “status” such as cardiac troponin, C-reactive protein and B-type natriuretic peptide. Further studies are necessary to extend the study to male animals and investigate the feasible value of sRAGE as a biomarker of cardiac aging and a measure of “biological age” in humans.

## Conclusion

Our observations demonstrate for the first time that RAGE is a regulator of intrinsic myocardial aging and point to circulating sRAGE as a cardio-protective molecule with anti-fibrotic activity. Its decline favors myocardial fibrosis progression that is critical for HF development. We suggest that the preservation of appropriate levels of sRAGE in the blood may be explored as a therapeutic strategy in order to promote successful aging and increase healthy lifespan.

## Supplementary Material

Supplementary figures and tables.

## Figures and Tables

**Table 1:** Body parameters and LV morphometric analysis

Variable	Young		MA		Old	
	WT	*Rage -/-*	WT	*Rage -/-*	WT	*Rage -/-*
Body Weight (BW, g)	19.42±1.17	20.83±1.67	31.46±4.21^‡‡‡^	33.20±4.70^‡‡‡^	30.64±3.23^‡‡‡^	35.06±5.06^‡‡‡†^
Tibia length (TL, mm)	17.71±0.87	17.04±0.81	18.41±0.6	18.86±0.67^‡‡‡^	19.50±0.47^‡‡‡§§^	19.00±0.64^‡‡‡^
Left Ventricle (LV) mass (mg)	76.66±7.98	93.33±12.35	116.70±19.86^‡‡‡^	143.50±19.62^‡‡‡††^	128.20±23.98^‡‡‡^	153.60±35.32^‡‡‡†^
LV/BW	4.02±0.37	4.61±0.54	3.74±0.56	4.45±0.80^†^	4.34±1.07	4.25±0.74
LV/TL	4.40±0.37	5.19±0.94	6.93±1.02^‡‡‡^	7.67±1.15^‡‡‡^	6.75±1.30^‡‡^	8.22±2.00^‡‡‡^

Data are represented as mean ± SD. ^‡‡^, P<0.01; ^‡‡‡^, P<0.001; 2-way ANOVA plus Bonferroni post-hoc test for multiple comparisons between MA or Old *vs* Young group.^ §§^, P<0.01; 2-way ANOVA plus Bonferroni post-hoc test for multiple comparisons between Old *vs* MA of the same genotype. ^†^, P<0.05;^ ††^, P<0.01; 2-way ANOVA plus Bonferroni post-hoc test for multiple comparisons between *Rage-/- vs* aged-matched WT group; n= 8-21.

**Table 2:** Correlation and multivariate regression analysis between serum sRAGE level and echocardiographic parameters in mice of different age

Variable	Correlation analysis			Multivariable regression analysis adjusted for age		
	r		P	β	SE	P
log LVESV (µL)		-0.219	0.305	-0.1488	0.1497	0.331
log LVEDV (µL)		-0.426	**0.038**	-0.1601	0.0569	**0.010**
log LVESD (mm)		-0.158	0.471	-0.0411	0.0607	0.507
LVEDD (mm)		-0.427	**0.042**	-0.5691	0.2096	**0.013**
SV (µL)		-0.465	**0.022**	-17.0588	5.0588	**0.003**
EF (%)		-0.070	0.746	-1.2416	4.2110	0.771
FS (%)		-0.075	0.727	-1.4153	4.5724	0.760
LVAWT, s (mm)		-0.284	0.189	-0.2170	0.1589	0.187
LVAWT, d (mm)		-0.144	0.511	-0.0677	0.1130	0.556
LVPWT, s (mm)		0.110	0.609	0.1415	0.2063	0.500
log LVPWT, d (mm)		0.311	0.139	0.2084	0.1233	0.106
LV mass (mg)		-0.033	0.878	0.9857	15.7960	0.951
						

In bold statistically significant P value. n = 23-24. LVESV: Left Ventricle end-systolic volume; LVEDV: Left Ventricle end-diastolic volume; LVESD: Left Ventricle end-systolic diameter; LVEDD: Left Ventricle end-diastolic diameter; SV: Stroke Volume; EF: Ejection Fraction; FS: Fractional Shortening; LVAWT, s: Left Ventricle Anterior Wall Thickness in systole; LVAWT, d: Left Ventricle Anterior Wall Thickness in diastole; LVPWT, s: Left Ventricle Posterior Wall Thickness in systole; LVPWT, d: Left Ventricle Posterior Wall Thickness in diastole.

## Abbreviations

α-SMA: α-Smooth Muscle Actin
ADAM10: Disintegrin and Metalloproteinase Domain-containing Protein 10
AGEs: Advanced Glycation End-Products
ANG: II Angiotensin II
ANKRD1: Ankyrin Repeat Domain 1
AT1: Angiotensin II Receptor Type 1
BNP: Brain Natriuretic Peptide
CAD: Coronary Artery Disease
CFs: Cardiac Fibroblasts
CM: Cardiomyocytes
cRAGE: cleaved RAGE
CTGF: Connective Tissue Growth Factor
CVD: Cardiovascular Diseases
ECM: Extracellular Matrix
EF: Ejection Fraction
esRAGE: endogenous secretory RAGE
FL-RAGE: Full-Length RAGE
FS: Fractional Shortening
hcFbs: human cardiac Fibroblasts
HF: Heart Failure
HMGB1: High Mobility Group Box-1
LV: Left Ventricle
LVEDD: Left Ventricle End-Diastolic Diameter
LVEDV: Left Ventricle End-Diastolic Volume
LVESD: Left Ventricle End-Systolic Diameter
LVESV: Left Ventricle End-Systolic Volume
LVPWT, d: Left Ventricle Posterior Wall Thickness in diastole
MAPKs: Mitogen-Activated Protein Kinases
MMPs: Matrix Metallopeptidases
MyoFbs: MyoFibroblasts
NF-κB: Nuclear Factor kappa-light-chain-enhancer of activated B cells
PPAR-γ: Peroxisome-Proliferator-Activated Receptor-γ
PRR: Pattern Recognition Receptor
RAGE: Receptor for Advanced Glycation End-Products
SMAD: Small Mother Against Decapentaplegic
sRAGE: soluble Receptor for Advanced Glycation End-Products
SV: Stroke Volume
TGFbetaR1: Transforming Growth Factor-beta Receptor 1
TGF-β1: Transforming Growth Factor-β1
WT: Wild Type
