# Supplementary material for: Soluble Receptor for Advanced Glycation End-products regulates age-associated Cardiac Fibrosis
Source: Int J Biol Sci. 2021 Jun 11;17(10):2399–416. doi: 10.7150/ijbs.56379 (PMC8315019; doi:10.7150/ijbs.56379)
Supplement: Supplementary file 1 — Supplementary figures and tables. [file ijbsv17p2399s1.pdf]

Soluble Receptor For Advanced Glycation End-products Regulates Age-associated Cardiac Fibrosis

Francesco Scavello[1,*], Filippo Zeni[1,*], Giuseppina Milano[2,§], Federica Macrì[1], Stefania Castiglione[1], Estella Zuccolo[1], Alessandro Scopece[2], , Giovanni Pezone[1], Calogero C. Tedesco[3], Patrizia Nigro[2], Genny Degani[4], Elisa Gambini[2], Fabrizio Veglia[3], Laura Popolo[4], Giulio Pompilio[2], Gualtiero I. Colombo[5], Marco E. Bianchi[6] and Angela Raucci[1,#]

1 Unit of Experimental Cardio-Oncology and Cardiovascular Aging, Centro Cardiologico Monzino-IRCCS, Milan, Italy. 2 Vascular Biology and Regenerative Medicine Unit, Centro Cardiologico Monzino-IRCCS, Milan, Italy. 3 Unit of Biostatistics, Centro Cardiologico Monzino-IRCCS, Milan, Italy. 4 Department of Biosciences, University of Milan, Milan, Italy. 5 Unit of Immunology and Functional Genomics, Centro Cardiologico Monzino-IRCCS. 6 Chromatin Dynamics Unit, San Raffaele University and IRCCS San Raffaele Hospital, Milan, Italy.

* Equal contribution

§ Department of Cœur-Vaisseaux, Laboratory of Cardiovascular Research, Centre Hospitalier Universitaire Vaudois, Lausanne, Switzerland.

#Corresponding author: Angela Raucci, PhD, Unit of Experimental Cardio-Oncology and Cardiovascular Aging, Centro Cardiologico Monzino, Via Carlo Parea 4, 20138, Milan, Italy; e-mail: araucci@ccfm.it; Tel. +39 0258002802; Fax +39 0258002342.

Supplementary Materials

Table S1. Patient general information and features of derived hcFbs.

**Patient ID**	**Age (years)**	**Gender**	**History**	**CD44, CD29, CD73, CD105, CD90**	**CD45, CD14, CD34, CD31, HLA-DR**
#1	34	F	Healthy	+	-
#2	50	F	Healthy	+	-
#3	74	F	Valvular Heart Disease	+	-

For isolation of hcFbs, right auricle was used from cadaveric donors (#1, #2) or donors undergone cardiac surgery (#3). Cells were characterized by flow cytometry for mesenchymal (CD44, CD29, CD73, CD105, CD90), inflammatory (CD45, CD14, CD34, CD31) and immunogenic (HLA-DR) markers. F= Female.

Table S2. Sequence of human (h) and mouse (m) forward and reverse primers used for qPCR.

Gene	Forward	Reverse
hCOL1A1	5’-TTTCAGTGGTTTGGATGGTG-3’	5’-ACCATCATTTCCACGAGCAC-3’
hCOL3A1	5’-CCCGGAAGTCAAGGAGAAAG-3’	5’-TCCCTGAGGTCCAGTTTCAC-3’
hACTA2	5’-ATCCTTCATCGGGATGGAGTCT-3’	5’-GGAGGGGCAATGATCTTGATCTT-3’
hCCN2	5’-AAGCTGCCCGGGAAATG-3	5’-TGGGCCAAACGTGTCTTC-3’
hTGFB1	5’-GTACCTGAACCCGTGTTGCT-3’	5’-CACAACTCCGGTGACATCAA-3’
hMMP2	5’-ACCCAGATGTGGCCAACTAC-3’	5’-GGTCACATCGCTCCAGACTT-3’
hHPRT1	5’-CCTGGCGTCGTGATTAGTGA-3’	5’-GCCTCCCATCTCCTTCATCA-3’
mCol1a1	5’-GGAGGGCGAGTGCTGTGCTTT-3’	5’-GGGACCAGGAGGACCAGGAAGT-3’
mActa2	5’-GACGCTGAAGTATCCGATAGAACACG-3’	5’-CACCATCTCCAGAGTCCAGCACAAT-3’
mCcn2	5’-AGAGTGGAGCGCCTGTTCTA-3’	5’-CCGCAGAACTTAGCCCTGTA-3’
mTgfb1	5’-AGCCCGAAGCGGACTACTAT-3’	5’-TCCACATGTTGCTCCACACT-3’
mNppb	5’-CTGAAGGTGCTGTCCCAGAT-3’	5’-CCTTGGTCCTTCAAGAGCTG-3’
mAnkrd1	5’-CGGACCTCAAGGTCAAGAAC-3’	5’-TGAGGCTGTCGAATATTGCTT-3’
mRAGE	5’-TCCTCAGGTCCACTGGATAAAG-3’	5’-TTCAGCTGGCCCCTCATCGCC-3’
mRAGE + mRAGE_v4	5’-TCCTCAGGTCCACTGGATAAAG-3’	5’-TGTGACCCTGATGCTGACAGG-3’
mGusb	5’-TATGGGCATTTGGAGGTGAT-3’	5’-GCTCTCCGACCACGTATTCT-3’
mLdha	5’-AGACAAACTCAAGGGCGAGA-3’	5’-CAGCTTGCAGTGTGGACTGT-3’
mPpih	5’-CTGGAGTCGCCAGTATTTACC-3’	5’-CTTTCCATCCAGCCAATCAC-3’
mHprt	5’-GGAGCGGTAGCACCTCCT-3’	5’-CCAAATCCTCGGCATAATGA-3’

Acta2=Alpha-actin-2/alpha smooth muscle actin; RAGE=Advanced glycosylation end-product receptor; Ankrd1=Ankyrin repeat domain 1; Ccn2=Cellular communication network factor 2/Connective Tissue Growth Factor; Col1a1=pro-Collagen type I alpha 1 chain; COL3A1=pro-Collagen type III alpha 1 chain; Gusb=Glucuronidase beta; Hprt=Hypoxanthine guanine phosphoribosyl transferase; Ldha=Lactate dehydrogenase A; MMP2=Matrix metalloproteinase 2; Nppb=Natriuretic peptide B; Ppih=Peptidylprolyl isomerase H; Tgfb=Transforming growth factor beta.

Table S3. Echocardiographic analysis of Middle-age WT animals after treatment with sRAGE.

Variable	CTRL	sRAGE
**LVESV (uL)**	9.01±1.17	8.78±1.14
**LVEDV (uL)**	43.70±5.57	48.89±3.48
**LVESD (mm)**	1.73±0.09	1.71±0.08
**LVEDD (mm)**	3.28±0.18	3.43±0.10
**EF (%)**	79.29±2.43	82.06±2.89
**FS (%)**	47.07±2.34	50.07±3.18
**SV (uL)**	34.69±5.00	40.11±3.79
**LVPWT, d (mm)**	0.81±0.12	0.86±0.23

LVESV= Left Ventricle end-systolic volume; LVEDV= Left Ventricle end-diastolic volume; LVESD= Left Ventricle end-systolic diameter; LVEDD= Left Ventricle end-diastolic diameter; SV= Stroke Volume; EF= Ejection Fraction; FS= Fractional Shortening; LVPWT, d = Left Ventricle Posterior Wall Thickness in diastole. CTRL = Control mice.

Supplementary Figures

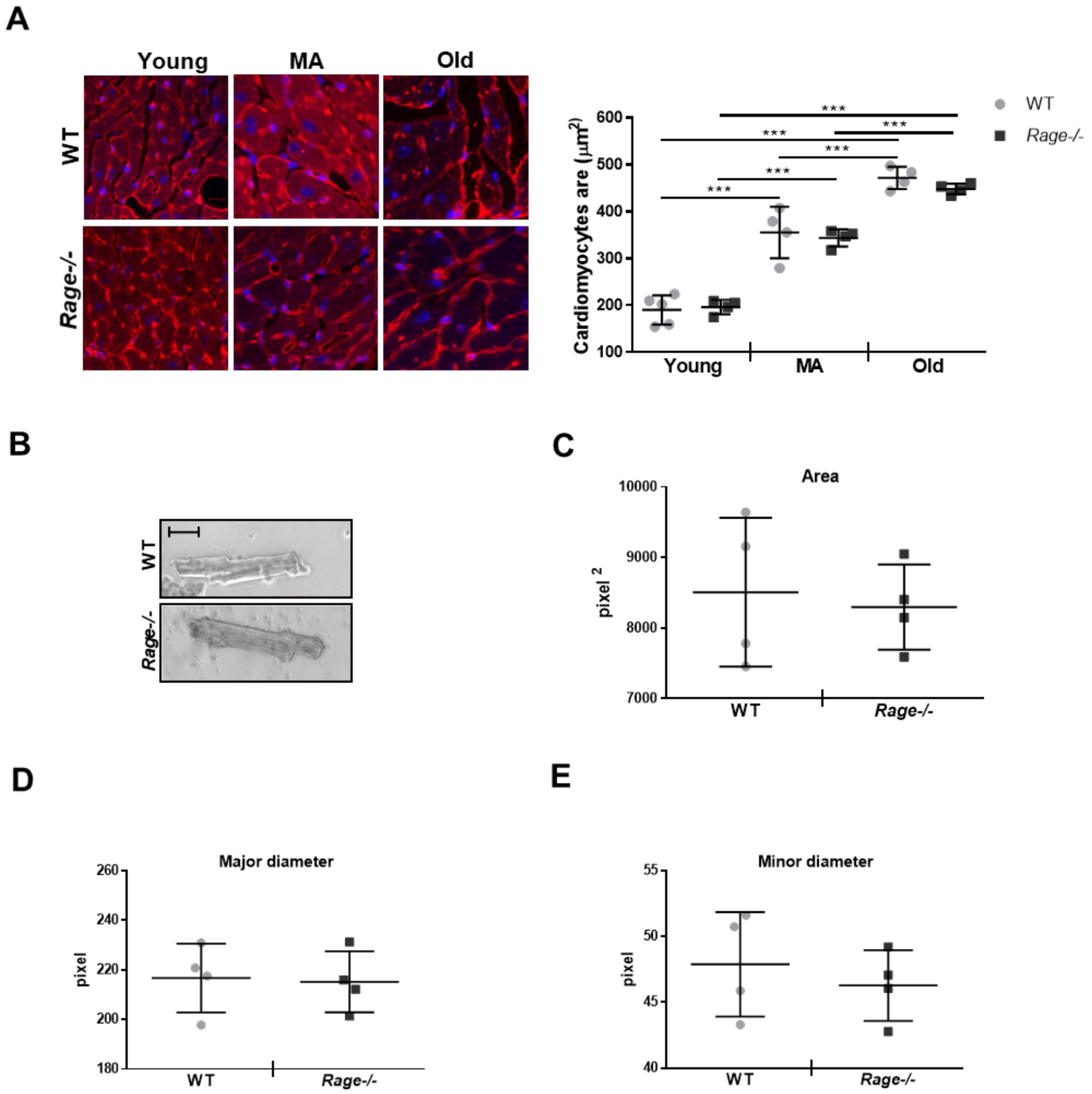

Figure S1. RAGE deficiency does not influence age-dependent cardiomyocytes area. (A) (Left panel) Representative images of cross-sectional areas of LV cardiomyocytes of Young, Middle-age (MA) and Old WT or *Rage-/-* mice stained with WGA (red). Nuclei are stained with Hoechst (blue). Bar, 10 µm. (Right panel) Quantification of CM area. Data are represented as mean ± SD (***, P<0.001; 2-way ANOVA plus Bonferroni post-hoc test for multiple comparisons; Young WT n=5, Young *Rage-/-* n=4, MA WT n=4, MA *Rage-/-* n=4, Old WT n=4, Old *Rage-/-* n=4). **(B)** Representative images of cardiomyocytes isolated from MA WT and *Rage-/-* mice. Bar, 50 pixel. **(C-E)** Measure of Area (c), Major (d) and Minor (e) diameter of isolated CM from MA animals. Data are represented as mean ± SD (t test; MA WT n=4, MA *Rage-/-* n=4).

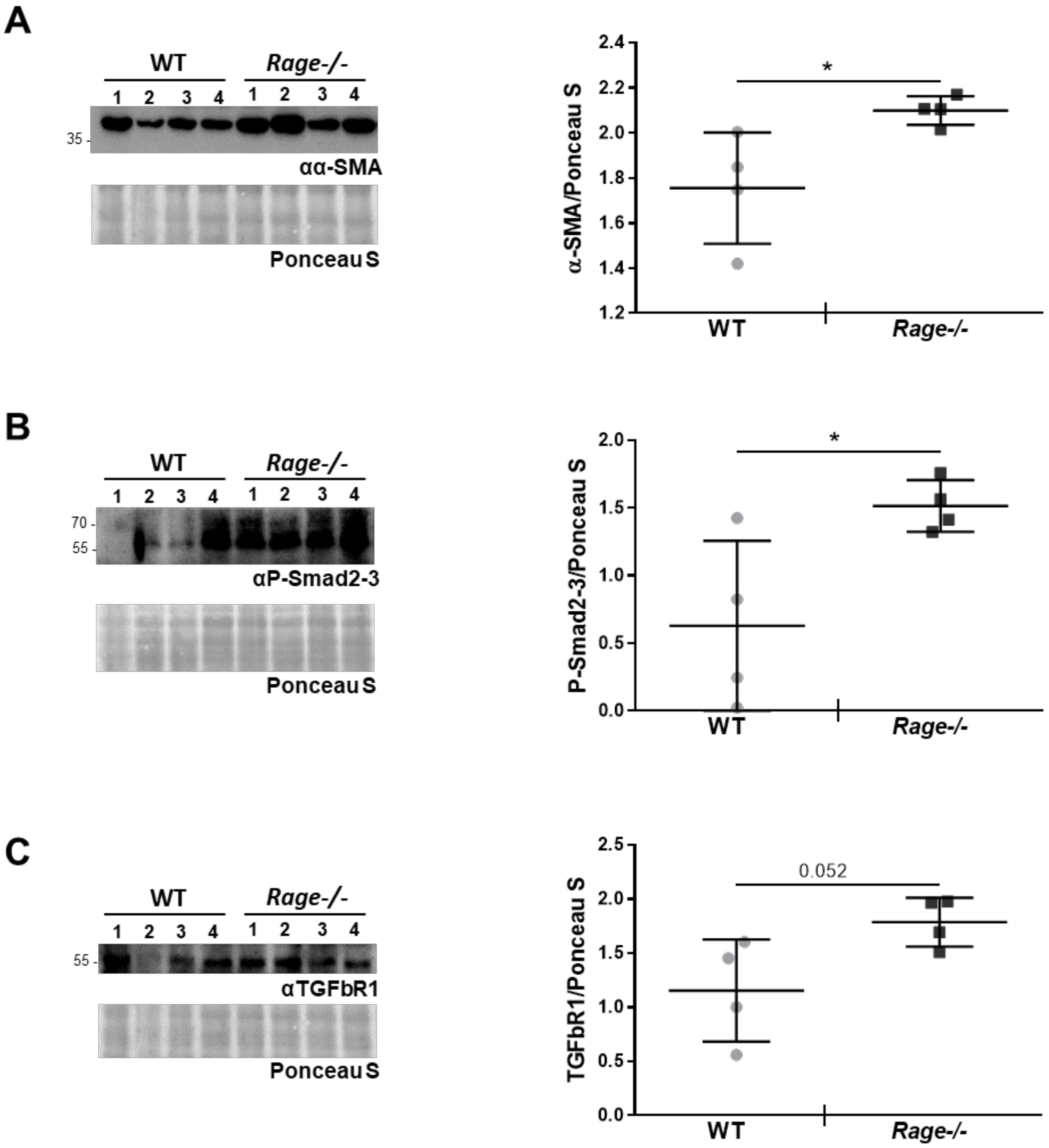

Figure S2. RAGE deficiency induces age-mediated pro-fibrotic protein expression. (A-C) (Left panels) Left Ventricle protein expression of α-SMA (A), P-Smad2-3 (B) and TGFbR1 (C) in Middle-age (MA) WT and *Rage-/-* mice. (Right panels) Quantification of Western Blot experiments. WT n=4, Rage-/- n=4. Each dot represents a mouse; mean ± SD are shown (*, $P<0.05$; t-test).

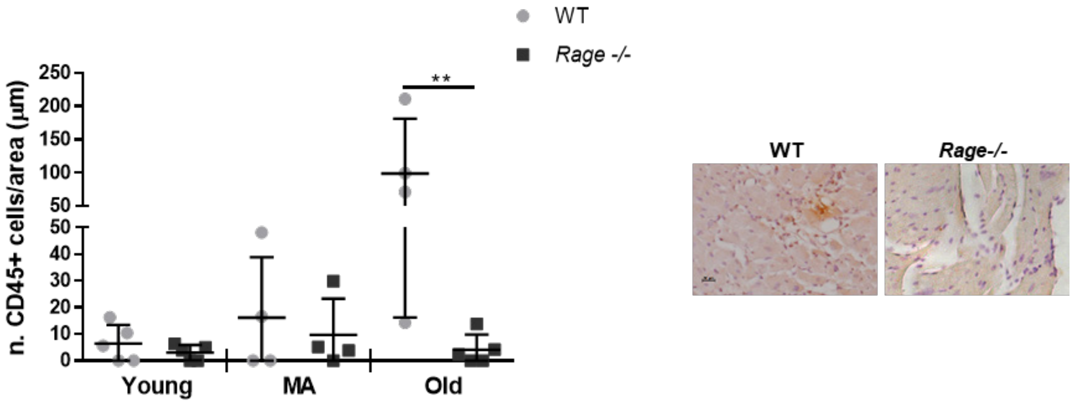

Figure S3. RAGE deficiency does not influence age-dependent cardiac inflammation. (Left panel) Quantification of CD45+ cells present in the heart mid-chamber sections of indicated groups of animals. (Right panel) Representative images of Old WT or *Rage-/-* mice stained with an antibody against CD45 antigen. Bar, 20 µm. Data are represented as mean ± SD (*, P<0.05; 2-way ANOVA plus Bonferroni post-hoc test for multiple comparisons; Young WT n=5, Young *Rage-/-* n=5, MA WT n=4, MA *Rage-/-* n=4, Old WT n=4, Old *Rage-/-* n=5).

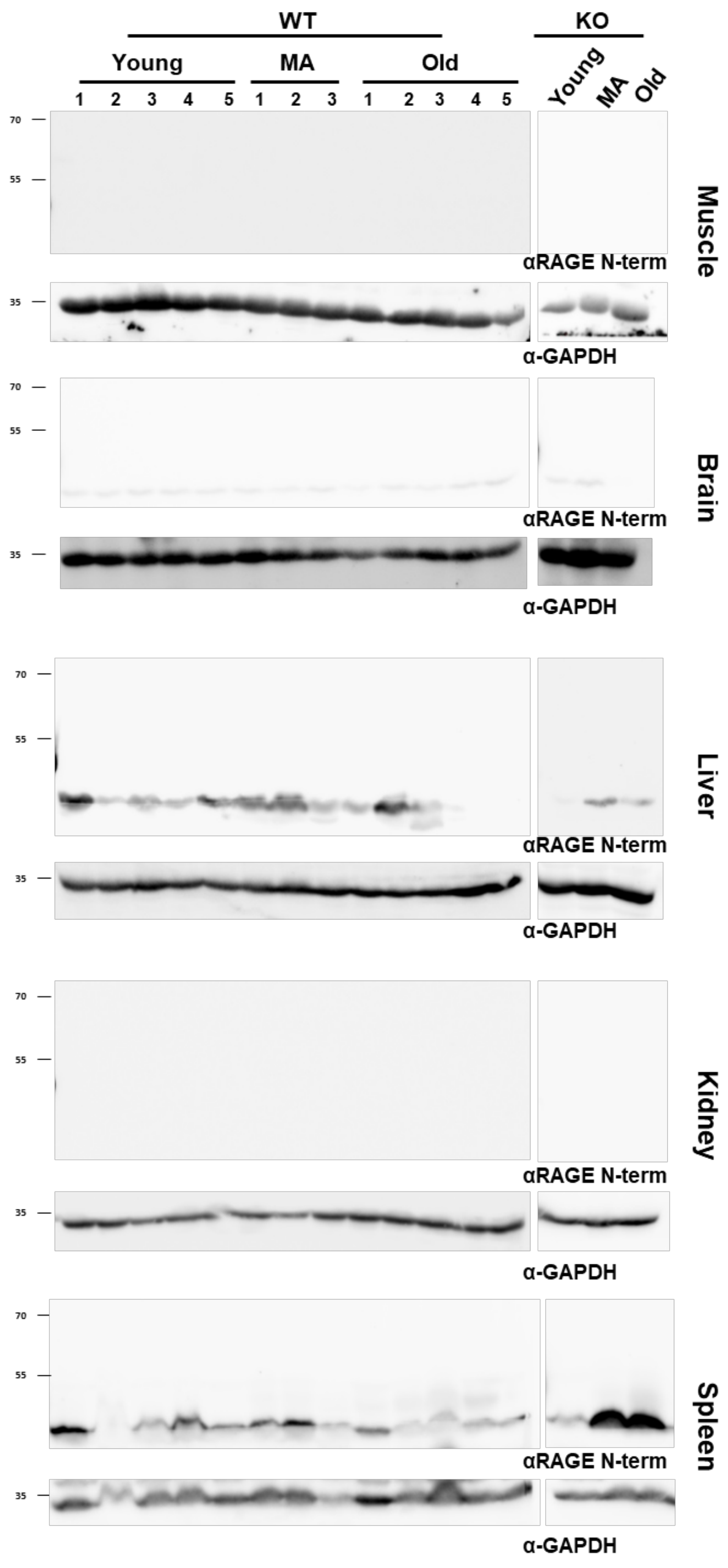

Figure S4. RAGE isoforms expression in several murine organs at different age. Forty µg of protein lysate of indicated organs isolated from Young, Middle-age (MA) and Old WT mice were probed with an antibody α-RAGE (RAGE N-term). Same amount of protein lysate from one Young, MA or Old *Rage-/-* mouse was loaded as negative control. Detection of GAPDH was used as loading control; n=3-5/group.

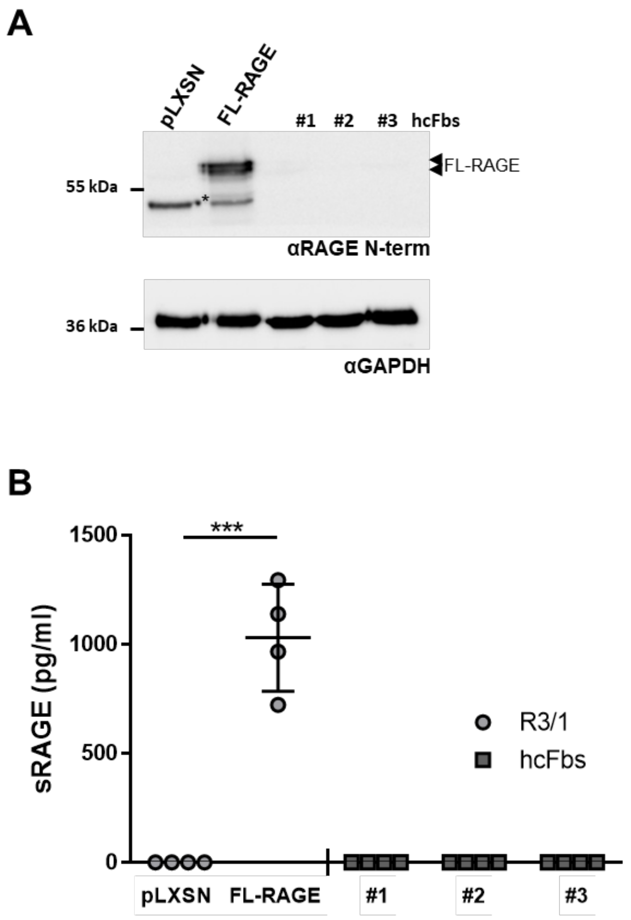

Figure S5. HcFbs do not express any isoforms of RAGE. (A) Expression of FL-RAGE in R3/1-pLXSN (pLXSN, negative control) and R3/1-FL-RAGE (FL-RAGE, positive control) cells or hcFbs isolated from three different donors (Supplementary Table 1), was determined by Western blot using 10 µg of protein lysate with a specific antibody raised against RAGE (αRAGE N-term). * indicates non-specific bands. GAPDH was used as normalizer. **(B)** Quantification of cRAGE in the supernatant of R3/1-pLXSN (pLXSN) and R3/1-FL-RAGE (FL-RAGE) cells or hcFbs isolated from three different donors 48 h after medium changing by means of ELISA assay. Data are expressed as mean ± SD (***, $P<0.001$; t test; n = 4).

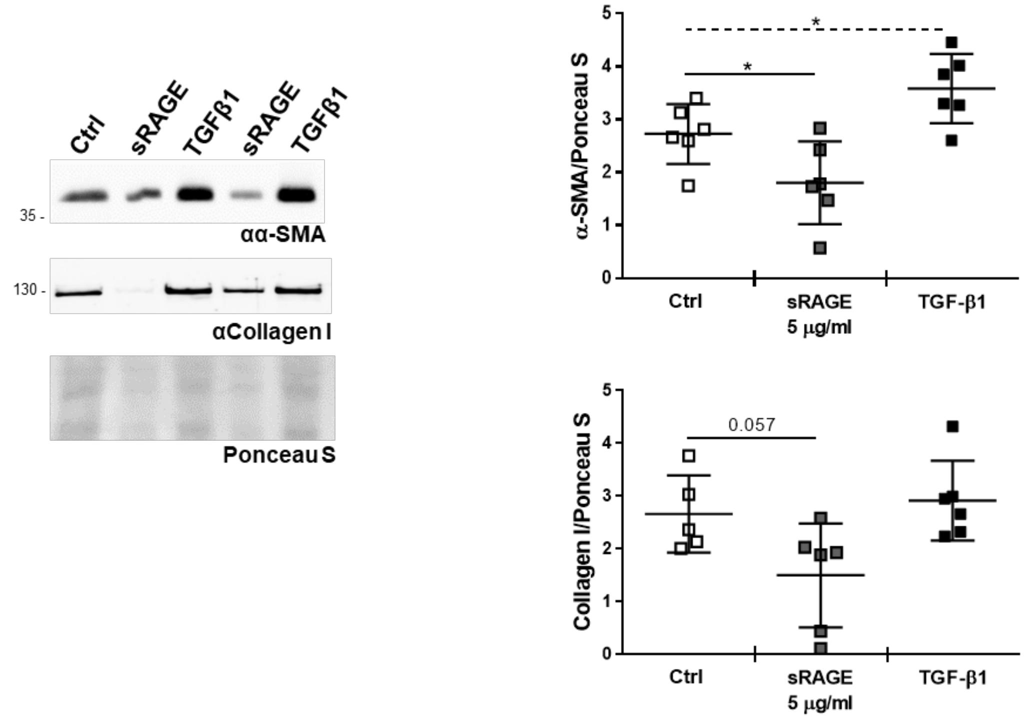

Figure S6. Recombinant sRAGE reduces α-SMA and Collagen I expression in hcFbs. HcFbs were stimulated with nothing (Ctrl) or 5 µg of sRAGE for 24 h. TGF-β1 (10 ng/ml) was used as positive control. Twenty µg of protein extracts were probed with an antibodies against α-SMA or Collagen I. (Left panels) Representative images of Western Blot analysis. (Right panels) Quantification of Western Blot experiments; (n=5-6/group). Each dot represents a biological replicate; mean ± SD are shown (*, P<0.05; continuous line: t-test between Ctrl and sRAGE; dotted line: t-test between Ctrl and TGF-β1).

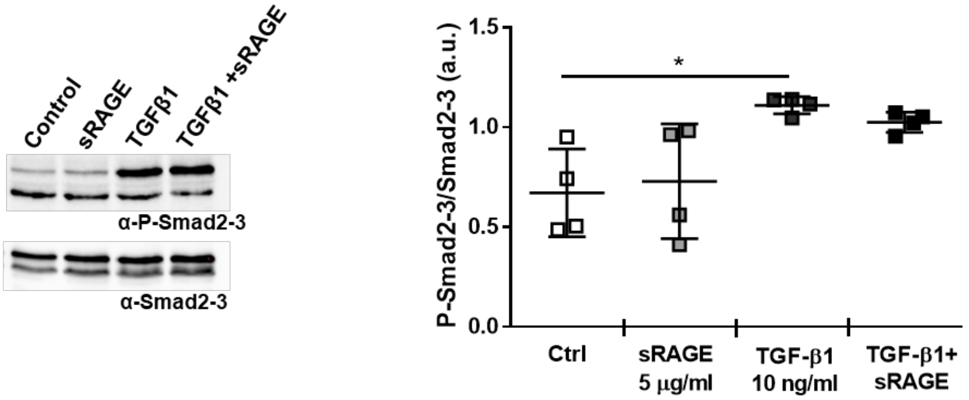

Figure S7. sRAGE does not counteract TGF-β1-dependent Smad2-3 activation in hcFbs. Cells were stimulated with normal medium, as negative control, sRAGE 5μg/mL, TGFβ1 10 ng/mL and combination of TGFβ1 10 ng/mL +sRAGE 5μg/mL for 20 minutes. Ten μg of protein extracts were probed with an antibody α-P-Smad2-3 or total α-Smad2-3. (Left panel) Representative images of Western Blot analysis. (Right panel) Quantification of Western Blot experiments; (n=4/group). Data are mean ± SD (*, $P<0.05$; 1-way ANOVA plus Bonferroni post-hoc test for multiple comparisons).

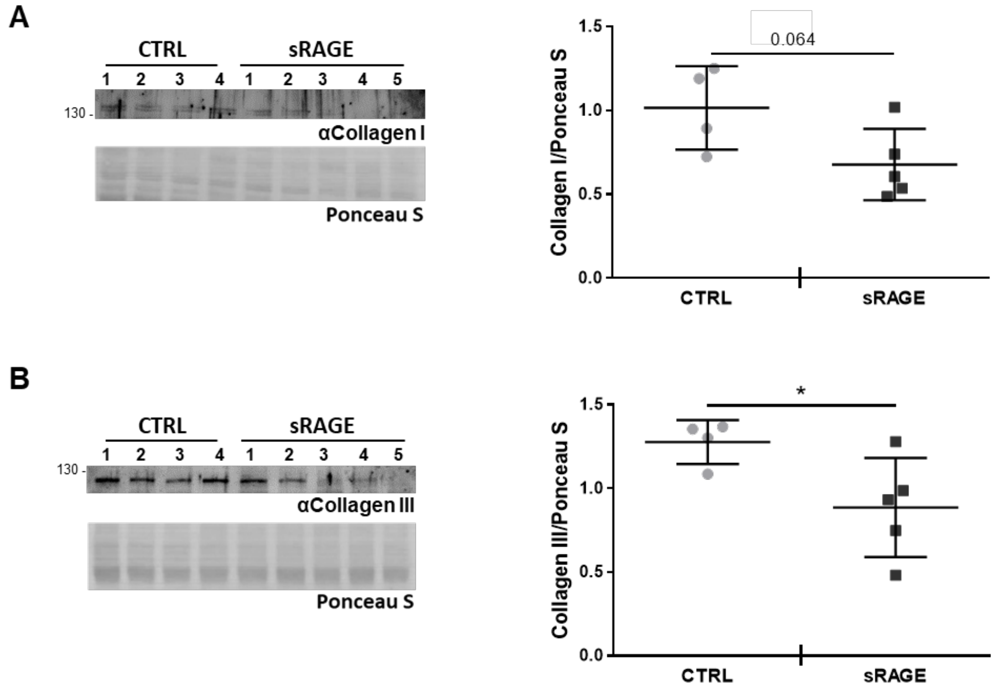

Figure S8. Recombinant sRAGE reduces Collagen I and III expression *in vivo*. Middle-age WT mice were injected daily with about 22 µg of recombinant murine sRAGE (sRAGE) for 8 days or equal volume of physiological salt solution (CTRL). **(A, B)** (Left panels) Western blot analysis of 35 µg of LV protein lysate probed with an antibody αCollagen I and αCollagen III. Red Ponceau staining (Ponceau S) was used as loading control. (Right panels) Quantification of Collagen I and Collagen III expression in the LV protein lysate. CTRL n=4, sRAGE n=5. Each dot represents a mouse; mean ± SD are shown (*, P<0.05; t test).
